# The relationship between emotional impulsivity (Urgency), aggression, and symptom dimensions in patients with borderline personality disorder

**DOI:** 10.1186/s40479-025-00292-5

**Published:** 2025-05-15

**Authors:** Sylvia Martin, Jonathan Del Monte, Richard Howard

**Affiliations:** 1 Center for Research and Bioethics, Uppsala University, Hursagatan 3, A 11 BMC, Uppsala, Sweden; 2 Psychology Department, Nîmes University, Nîmes, France; 3 Institute of Mental Health, Nottingham, UK

**Keywords:** Borderline personality disorder, Aggression, Anger, Irritability, Impulsivity, UPPS, Urgency, Positive Urgency, Negative Urgency

## Abstract

**Background:**

A hallmark of borderline personality disorder (BPD) is a disposition to anger, irritability and aggression. High impulsivity, particularly high emotional impulsivity (urgency), has been associated with aggression in BPD patients.

**Aims:**

This study aimed to explore, in a sample of patients with BPD, the subtleties of the relationship between borderline symptomatology, different facets of impulsivity, and an aggressive disposition.

**Methods:**

Two hundred and twenty patients with a DSM-5 (Sect. 2) diagnosis of BPD were assessed on measures of impulsivity (UPPS model), aggression (Brief Aggression Questionnaire, BAQ-12) and borderline symptoms (Borderline Personality Questionnaire, BPQ).

**Results:**

Results showed: (i) there was a close relationship between BPD symptomatology and an aggressive predisposition measured by BAQ-12; (ii) emptiness and intense anger were the BPD symptom dimensions most significantly associated with aggression (iii) both negative and positive urgency, and to a lesser extent lack of premeditation and sensation seeking, mediated the relationship between borderline symptom dimensions and aggression.

**Discussion & conclusion:**

Results suggest a close relationship between almost all dimensions of BPD, but especially anger, and impulsive aggression. They further suggest that urgency, particularly negative urgency, mediates this relationship. Future studies will need to parse aggression into motivationally distinct types.

**Supplementary Information:**

The online version contains supplementary material available at 10.1186/s40479-025-00292-5.

## Introduction

### Borderline personality disorder (BPD)

Historically BPD has been regarded as one of the most prevalent and impactful personality disorders within psychiatric populations, affecting approximately 10% of psychiatric outpatients and 15–25% of hospitalized patients [1]. Impulsive and aggressive behaviours are characteristic of patients diagnosed with BPD. Impulsive behaviour in areas such as spending, sex, substance abuse and reckless driving is a diagnostic criterion in DSM-5 [2], while emotional impulsiveness (“A tendency to act rashly in states of high negative affect, leading to potentially self-damaging behaviours”) is emphasized in the borderline pattern described in the 11th iteration of the International Classification of Diseases [3]. However, precisely how BPD, impulsivity and aggression are related remains unclear.

BPD is a very heterogeneous disorder that is rampantly comorbid with other disorders [4]. Some authors (e.g [5]). have contested its status as a personality disorder, while others (e.g [6]). have suggested that BPD reflects overall severity of personality dysfunction rather than being a distinct clinical entity. Evidence suggests that impulsivity and aggression may be especially characteristic of particular subtypes of BPD, e.g. the ‘externalizing’ cluster identified by Oladottir et al. the ‘impulsive’ subgroup identified among borderline patients by Gamache et al. [7, 8].

### Emotional impulsivity (urgency) in BPD

A large body of research underscores the central role of impulsivity in the symptomatology and treatment challenges of BPD [9–13]. Particularly implicated in BPD is emotional impulsivity or ‘Urgency’ – the tendency to act rashly in the context of strong emotion, typically operationalized by Urgency scales from the UPPS model [14]. However, Urgency has been found to be associated with a broad spectrum of psychopathology [15], and with most PDs, particularly those in Cluster B (antisocial, borderline, narcissistic and histrionic PDs) [16]. Although sub-divided in the UPPS model into positive and negative scales, Urgency is thought to reflect a general tendency to act rashly when faced with intense emotional states, whether these are positive or negative [17, 18].

### Urgency, anger and aggression

No less heterogeneous than the borderline construct is the construct of aggression itself. Aggression has traditionally been parsed either by the form it can take (e.g. covert vs. overt, verbal vs. physical) or, in bimodal models, as reactive/impulsive vs. proactive/premeditated. Bimodal models arguably fail to account for the motivational heterogeneity of aggression, a problem addressed by the Quadripartite Violence Typology (QVT: [19, 20]). QVT is an extension and elaboration of the bipolar model, addressing its key deficiencies and arguably providing a more nuanced and motivationally enriched conceptualization of aggression. The intersection of two dimensions, *appetitive vs. aversive* and *impulsive vs. controlled*, yields four motivationally distinct aggression types (thrill-Seeking, explosive, coercive and vengeful) that have been validated in studies using the Angry Aggression Scales to operationalize the four types [21–23].

The literature supports a strong relationship between UPPS urgency, anger and aggression. Anger dysregulation linked to high urgency in BPD patients likely accounts for their high level of aggression [24, 25]. In a metanalysis Bresin identified the UPPS facets of urgency and lack of premeditation as being most closely associated with aggression [4]. In a study by Værøy et al. that compared violent prison inmates with controls, differences in impulsive and aggressive behaviours were largely attributable to higher urgency scores in the prison inmates [26].

It remains far from clear how symptoms of anger and irritability in BPD are linked to aggression. A study by Gröndal et al. [27] cast significant light on this question. They found that high levels of irritability predicted increased anger for participants with average to high levels of UPPS Urgency [27]. One possible interpretation of this finding was that urgency plays an important role in regulating the threshold between irritability and anger. In those BPD patients with higher levels of Urgency, feelings of irritability may more easily translate into anger, and hence into a readiness for aggression.

Results of recent studies that have used ecological momentary assessment (EMA) to monitor participants’ emotions and behaviours in their daily lives (e.g [28, 29]. suggest, first, that that in people with BPD, aggression is linked to high levels of, and fluctuations in, anger, which in turn are sensitive to specific interpersonal triggers (particularly perceived rejection); and second, that BPD symptoms and emotional impulsiveness (urgency) amplifies or sensitizes the rejection → anger arousal → aggression pathway.

### The current study

**Fig. 1:**
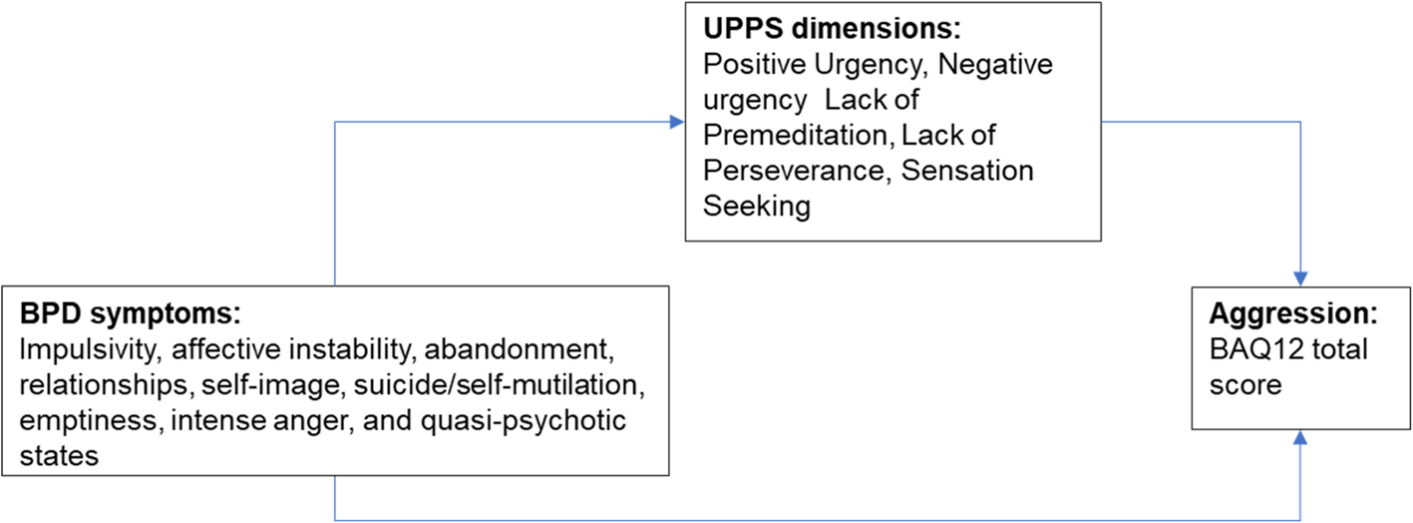
Model of predicted relationship between BPD symptoms, impulsivity and aggression

In the present study we aimed to explore, in a sample of patients with BPD, the subtleties of the relationship between borderline symptomatology, different facets of impulsivity, and an aggressive disposition. Specifically, we investigated: (i) which BPD symptom dimensions predicted aggression; and – assuming a link could be demonstrated between BPD symptoms and aggression – (ii) whether some facet or facets of UPPS impulsivity mediated the relationship between BPD symptoms and aggression. We tested a model of predicted relationships between BPD symptom dimensions, impulsivity and aggression shown in Fig. 1 below.

## Materials and methods

### Sample characteristics

Two hundred and twenty patients diagnosed with BPD (mean age = 37.06 years SD = 12.64, 16 males and 204 females) were recruited from the University hospital and a private psychiatric clinic with attached day-care unit. The mean time since first diagnosis was 35.94 months (SD = 59.21). Patients were included in the study provided they: (i) met DSM-5 criteria for BPD (at least 5 of 9 criteria); (ii) did not suffer from a comorbid Cluster B personality disorder; (iii) were in a stable phase of their illness (no change in housing or hospitalization in the previous month); Patients were excluded from the study if they had: (a) a known neurological disease; (b) a developmental disability; (c) a current substance use disorder; (e) a comorbid affective disorder (depression, bipolar disorder). All participants were proficient in the French language, had a normal or corrected-to-normal vision, were naïve about the study’s purpose, and gave their written informed consent to participate in the study. Data were collected during the period 2015 to 2019. Ethical approval was deemed unnecessary by Nîmes University Ethical Board since, at the start date for data collection, studies that did not involve a clinical intervention were exempt from requiring ethical approval. All patients fully completed the questionnaires detailed below.

### Measures 

#### Borderline personality questionnaire

We used the French version of the Borderline Personality Questionnaire (BPQ), which has a Cronbach’s α of 0.93 [30]. The BPQ is an 80-item questionnaire giving scores on dimensions corresponding to the following DSM-5 BPD criteria: impulsivity, affective instability, abandonment fears, disturbed relationships, disturbed self-concept, suicide/self-harm, emptiness, intense anger, and quasi-psychotic states [31]. BPQ shows good agreement with a BPD diagnosis using the Structured Clinical Interview for DSM-IV Axis II disorders (SCID-II) [32], The questions are answered true/false. Analyses showed high internal consistency (α = 0.84), good test-retest reliability (*r* = .77), significant discrimination of BPD from schizotypal personality disorder (*r* = − .31; *p* < .05), significant convergence with SCID-II (*r* = .72) and significant predictive validity of psychiatric diagnosis (*p* < .01) [33].

#### Impulsive behavior scale - short version (UPPS-S)

UPPS-S is a self-report scale comprising 20 items that assess five facets of impulsivity: (a) negative urgency: impulsive behaviour arising in the context of heightened negative affect (e.g., “When I am upset I often act without thinking”); (b) positive urgency; impulsive behaviour arising in the context of heightened positive affect e.g., “When I am really excited, I tend not to think on the consequences of my actions”); (c) lack of premeditation e.g., “Before making up my mind, I consider all the advantages and disadvantages” (d) lack of perseverance e.g., “I finish what I start” (reverse scored); and (e) sensation seeking e.g., “I sometimes like doing things that are a bit frightening”. The items were scored using a 4-point Likert scale (1 = strongly disagree to 4 = strongly agree). The present study used the French translation of the UPPS-S [34] whose psychometric validity and reliability have been confirmed across multiple languages, countries and gender identities [35]. Recent exploration measured each subscale mean score for psychiatric disorders that ranged from 9.85 (3.2) to 13.02 (2.86) for negative urgency, from 10.43 (2.84) to 12.56 (3.00) for positive urgency, from 8.77 (2.91) to 11.14 (2.92) for sensation seeking, from 7.26(2.69) to 8.25 (2.86) for Lack of Premeditation and from 7.12 (2.45) to 9.03 (3.21) for Lack of perseverance [36]. We will use the mean score reported for personality disorder as a reference score for moderate impulsivity with Negative urgency 8.49 (0.31), for Positive Urgency 7.26 (0.26), Lack of Premeditation 7.27 (0.21), Lack of Perseverance 7.36 (0.22), Sensation Seeking 10.22 (0.30).

#### Brief aggression questionnaire (BAQ12)

We used the French version [37] of the Brief Aggression Questionnaire (BAQ12) developed by Webster et al. [38]. BAQ12 comprises 12 items and measures four correlated dimensions (physical, verbal, emotional and cognitive) that reflect a person’s overall predisposition towards aggression. Items were answered using a Likert scale ranging from 1 (not at all like me) to 6 (completely like me), yielding a maximum total score of 72. We used the total score as our criterion measure of aggression. The Cronbach alpha’s is 0.79 for the BAQ12 Total score.

### Statistical analysis

Statistical analyses were run in SPSS 28.0.1.0 (142). As our data were normally distributed, we used parametric analysis. Relationships between BPQ, BAQ12, and impulsivity scores were first explored using Pearson parametric correlations. Multiple linear regression analyses were then applied to predict BAQ12 total score from BPQ dimensions and UPPS impulsivity facets. Finally, mediation analyses were carried out using PROCES 3.4. to test whether impulsivity measures, particularly urgency, significantly mediated the effects of BPQ dimensions on BAQ12 aggression. Significance level was set to *p* <.05.

## Results

### Descriptive analysis

Descriptive data showing mean scores for all measures are shown in Table S1 in supplementary information.

### Correlations

Correlations between variables are shown in Table 1. Both negative and positive urgency correlated significantly with BAQ12 and BPQ total. Negative urgency correlated significantly with all but one (suicidality/self-harm) of the individual BPQ dimensions, while positive urgency correlated significantly with all but 3 BPQ dimensions (suicidality/self-harm, disturbed self and emptiness). UPPS lack of premeditation and sensation seeking also correlated significantly with a majority of BPQ dimensions. BAQ12 correlated with all UPPS dimensions except lack of perseverance, and with all BPQ dimensions except suicide/self-harm.

**Table 1:** Correlation analysis

Variables	Negative urgency	Positive Urgency	Lack of Premeditation	Lack of Perseverance	Sensation seeking	UPPS	BAQ12
Impulsivity	0.367^**^	0.545^**^	0.463^**^	0.269^**^	0.305^**^	0.638^**^	0.65**
Affect Instability	0.402^**^	0.319^**^	0.348^**^	0.094	0.140	0.418^**^	0.413**
Abandonment fears	0.389^**^	0.215^**^	0.146^*^	0.053	0.162^*^	0.310^**^	0.415**
Relationship disturbance	0.400^**^	0.250^**^	0.187^*^	0.073	0.083	0.315^**^	0.329**
Self image	0.233^**^	0.109	0.280^**^	0.219^**^	− 0.036	0.259^**^	0.259**
Suicide/self-harm	0.058	0.018	− 0.120	− 0.193^**^	0.183^*^	− 0.017	0.084
Emptiness	0.295^**^	0.088	0.184^*^	0.289^**^	− 0.008	0.276^**^	0.424**
Intense Anger	0.563^**^	0.482^**^	0.237^**^	0.092	0.281^**^	0.531^**^	0.633**
QuasiPsychotic	0.350^**^	0.307^**^	0.112	0.040	0.296^**^	0.361^**^	0.333**
BPQTot	0.568^**^	0.426^**^	0.331^**^	0.168^*^	0.254^**^	0.564^**^	0.604**
BAQ12	0.561^**^	0.411^**^	0.255^**^	NS	0.256^**^	0.519^**^	-

Note: *=*p* <.05; **=*p* <.01; ***=*p* <.001

### Regression

We ran a linear regression analysis to test which UPPS facets predicted BAQ12 total score. Negative urgency was the strongest predictor (Beta = 0.453, *p* = .000) and positive urgency the second strongest (beta = 0.183, *p* = .024) (See Table 2) with R^2^ = 0.354 and F = 23.585. A second linear regression tested the prediction of BAQ12 from BPQ dimensions. Intense Anger was the strongest predictor (beta = 0.486, *p* = .000) and Emptiness the second strongest (beta = 0.271, *p* = .006) (see Table 3) with R^2^ = 0.501 and F = 13.835.

**Table 2:** Predicting BAQ12 Score from Impulsivity dimensions

	Non Standardized Coefficients		Standardized Coefficients	Significance
Model	B	Standard Error	Bêta	*p*
(Constant)	4.694	4.476		.206
Negative Urgency	1.912	.341	.453***	.000
Positive urgency	.792	.346	.183*	.024
lack of Premeditation	.376	.286	.097	.191
Adjusted R²		.339		
F		23.585		

Note: Dependant variable: BAQ12; *=*p*<.05; **=*p*<.01;***=*p*<.005

**Table 3:** Predicting BAQ12 from BPQ subdimensions

Variables	Non Standardized Coefficients		Standardized Coefficients	Significance
Model	B	Standard Error	Bêta	*p*
(Constant)	16.702	2.948		.000
Impulsivity	.47	.451	.008	.918
Affective instability	.538	.357	.113	.145
Abandonment	.483	.489	.094	.325
Relationships	-.208	.412	-.044	.614
Self Image	-.181	.506	-.033	.721
Suicide	-.404	.341	-.085	.238
Emptiness	1.201	.429	271**	.006
Intense Anger	1.977	.356	.468***	.000
Quasi Psychotic	.566	.434	.093	.94
Adjusted R²		.465		
F		13.835		

Note: Dependant variable: BAQ12; *=*p*<.05; **=*p*<.01;***=*p*<.005

### Mediation analysis

**Fig. 2:**
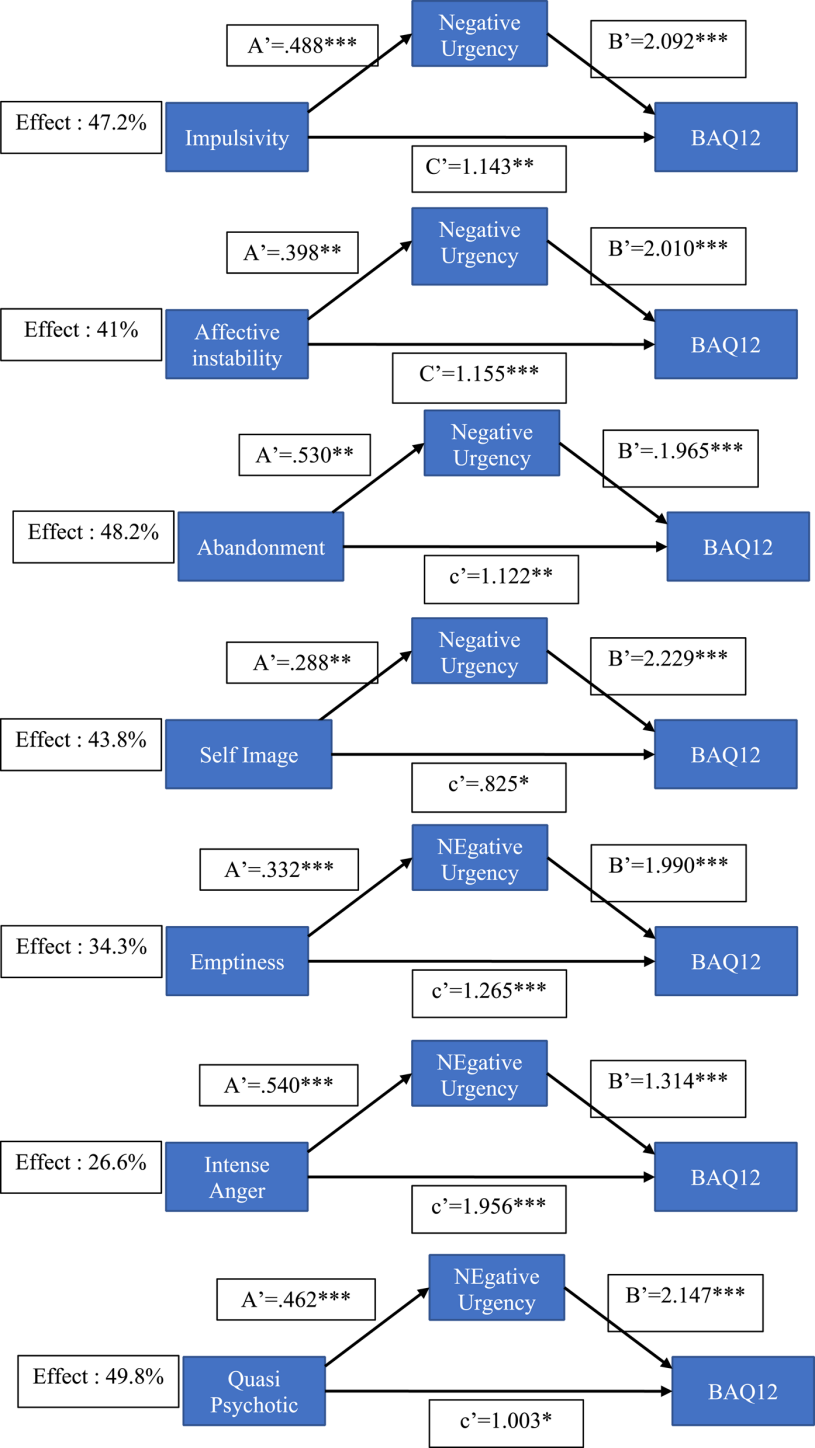
Mediations models representations with negative urgency. *p* < 05*, *p* < .005 ** and *p* < .001***

**Fig. 3:**
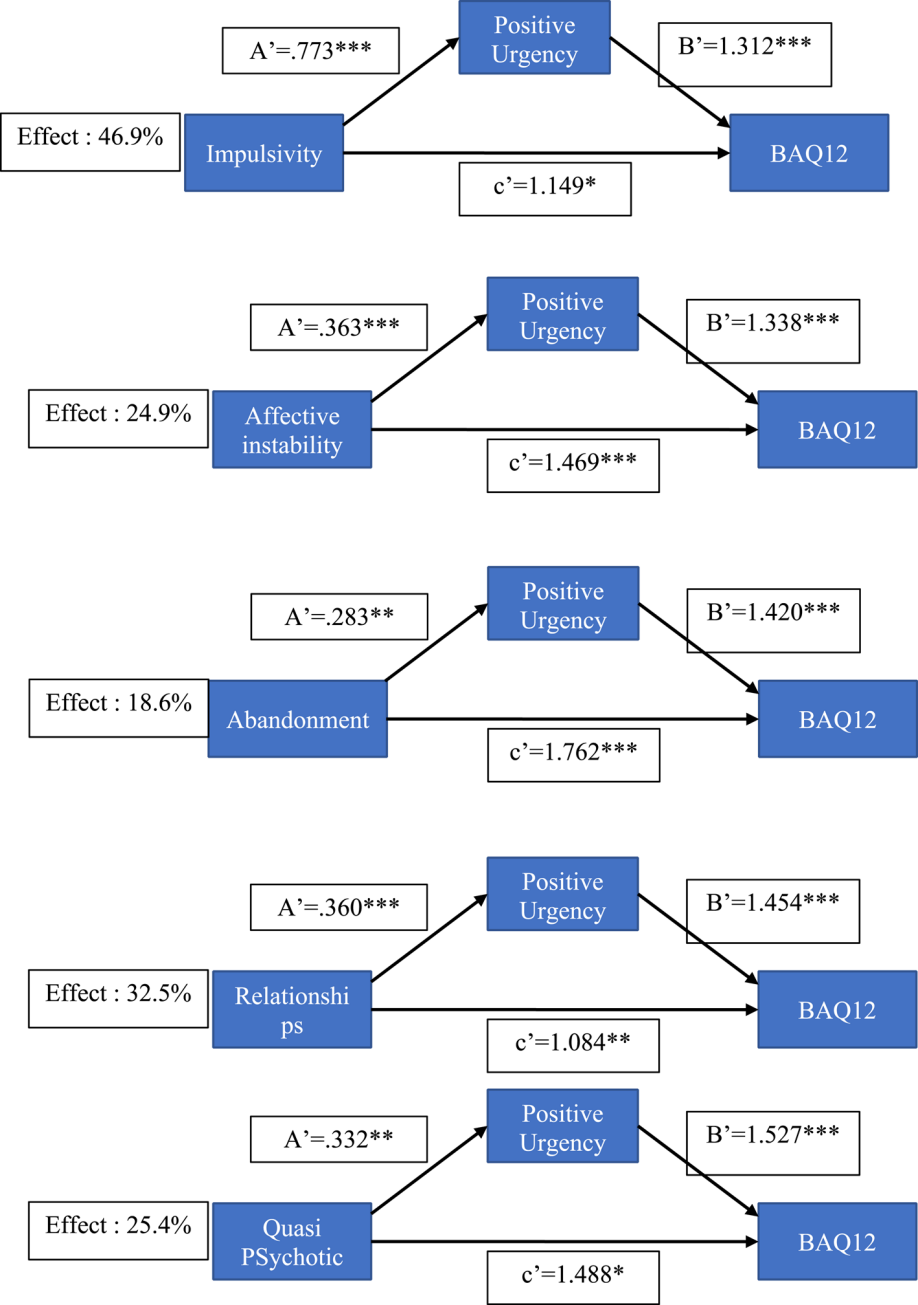
Mediation models with positive urgency

**Fig. 4:**
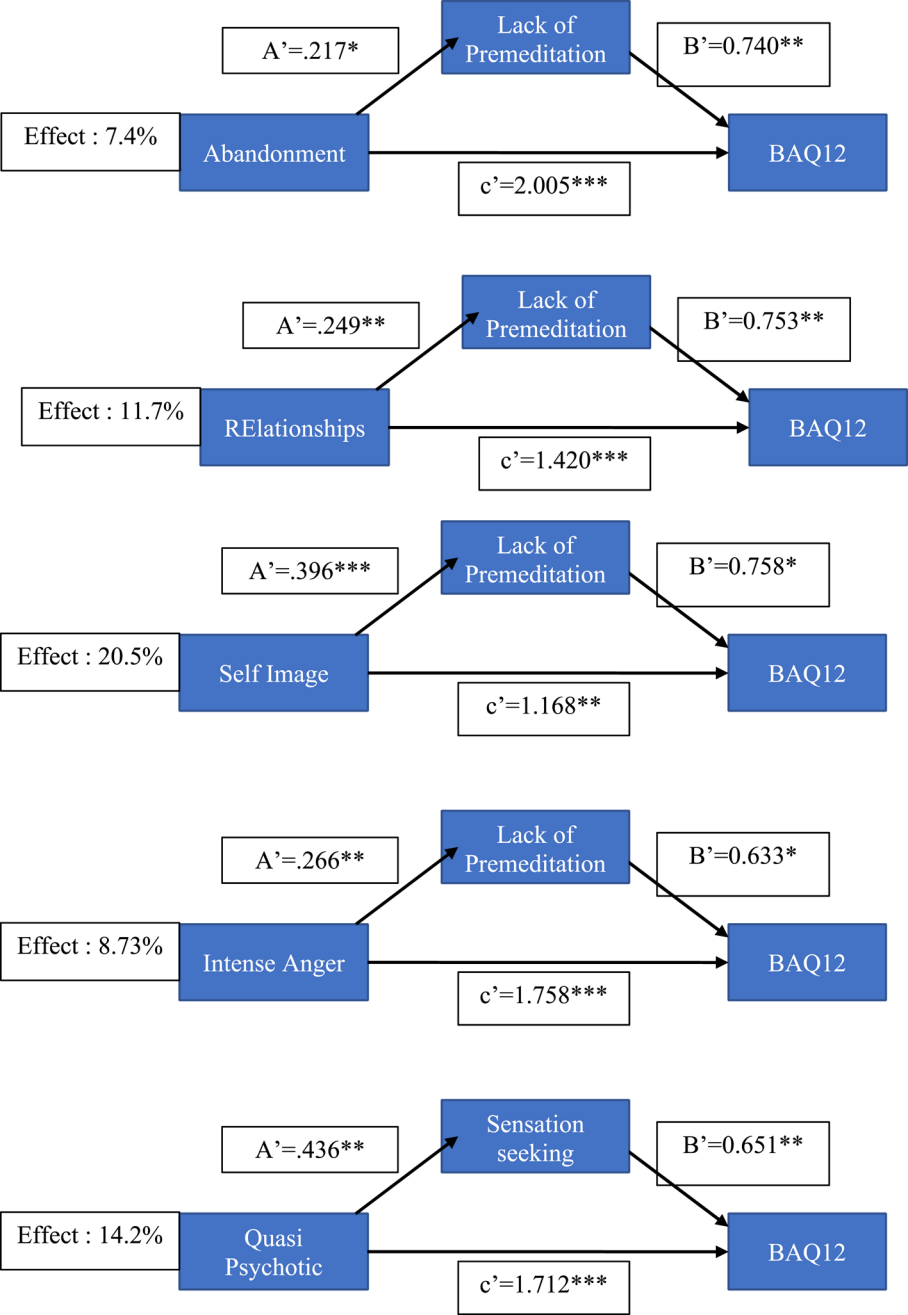
Mediation models with lack of premeditation and sensation seeking

We tested the degree to which UPPS impulsivity facets mediated the effects of BPD symptoms (BPQ) on aggression (BAQ12), with mediation expressed as a percentage of the total (direct plus mediated) effect. Significant mediation effects (see Figs. 2, 3 and 4 for details; supplementary file for all analysis) were found for negative urgency, positive urgency, lack of premeditation, and sensation seeking. Bonferroni correction was applied to ensure significance of mediation (Bonferroni alpha = 0.01) and all total estimates remained significant. The main results can be summarized as follows. Negative urgency mediated approaching 50% of the total effect, ranging from 27% for BPQ anger to 50% for BPQ quasi-psychotic. Positive urgency also showed a significant mediation effect, ranging from 19% for BPQ abandonment to 47% for BPQ impulsivity. Lack of premeditation showed a significant, albeit weaker, mediation effect that ranged from 7.4% (abandonment) to 20.5% (self-disturbance). A significant mediating effect of sensation seeking (14%) was found only for the relationship between BPQ quasi-psychotic and BAQ-12.

## Discussion

### Aggression

BAQ12 (overall aggression) correlated highly and significantly with both BPQ and UPPS measures of impulsivity, suggesting that overall aggression primarily measures impulsive aggression rather than controlled or premeditated aggression. The significant association found between overall aggression and borderline symptomatology is consistent with aggression in borderline patients being principally impulsive and reactive [39]. Results of regression analysis indicated that overall aggression could be strongly predicted by negative urgency and to a lesser degree by positive urgency. These results partly concur with a meta-analysis conducted by Bresin (2019) but unlike Bresin (2019), we did not find that lack of premeditation predicted aggression [4].

It is important to distinguish, as in the quadripartite violence typology [19] between aggression that is appetitively (vs. aversively) motivated and is impulsive (vs. premeditated). Bant et al. [40] found that negative urgency was significantly associated with both premeditated (motivated by a desire for revenge) and impulsive (motivated by a desire to remove an immediate social threat) types of aversively motivated aggression. This suggests that rather than being linked to impulsive aggression per se, negative urgency is more closely related to the experience of negative affect that accompanies both impulsive and premeditated types of aggression when they are aversively motivated. A further implication is that it is the negative affect associated with negative urgency that in part accounts for its ability to predict aggression, regardless of whether the aggression is impulsive or premeditated.

### Dimensions of borderline pathology

With the exception of self-harm/suicidality, all BPQ dimensions, as well as the BPQ total, were strongly and significantly correlated with overall aggression. Regression analysis indicated that among BPQ borderline dimensions, anger and emptiness were positive and significant predictor of overall aggression.

Despite being positively associated with aggression in the univariate analysis, emptiness emerged as a negative predictor of aggression in the multivariate analysis. Internalizing symptoms of borderline PD, including emptiness, load on a general dimension of PD labelled “Anxious-Inhibited” [41]. In contrast, externalizing symptoms of borderline PD, intense anger and impulsivity, load on a factor labelled “Acting Out”. In a similar vein, Cardona et al. [42] reported that BPD symptoms could be subsumed under two separate and independent factors, one reflecting externalizing factors (interpersonal Instability and hostility/impulsivity), the other reflecting internalizing factors: negative emotionality and negative affectivity. In their study that examined sexual and non-sexual aggression, Cardona et al. [42] found that neither internalizing factor added to the prediction of either type of aggression, sexual or non-sexual. They concluded that internalizing aspects of borderline PD such as emptiness do not appear to be independent predictors of aggression. Our results are consistent with this, and even suggest an inverse relationship between internalizing features of borderline PD and aggression. This may be because such internalizing features are associated with a hostile but *submissive* interpersonal style [41]. Submissiveness would seem to be antithetical to aggression, even when hostile feelings are experienced vis-à-vis others. Indeed, submissiveness measured with PID-5 in a mixed sample of personality disordered patients was reported to be a negative predictor of aggression [43].

With the exception of suicide/self-harm, all borderline symptom dimensions were correlated with UPPS impulsivity measures, most strongly with negative and positive urgency but also with lack of premeditation and sensation seeking. Self-harm/suicidality was positively correlated with sensation seeking, consistent with borderline patients’ verbal reports that they attempted to generate alternate affects to emptiness through impulsive behaviours such as self-harm [44]. Consistent with this, Bant et al. [23] found that impulsive aggression was associated with both positive and negative urgency. Recent studies show that considering negative and positive urgency separately has limited conceptual and methodological value [17]. It could be that negative/positive urgency is thus more reflective of the tendency to act impulsively while in an intense emotional state, irrespective of whether that state is positive or negative in valence. Interestingly, in our study positive urgency correlated more strongly with BPQ impulsivity than did negative urgency. These results support the proposal that sensation seeking is a significant aspect of borderline patients’ impulsive behaviour [45, 46]. The latter authors suggested that because of suboptimal arousal, BPD patients might have the tendency to approach exciting stimuli in an impulsive manner, resulting in highly dysfunctional behaviors.

### Mediation analysis

Mediation analysis demonstrated that both negative and positive urgency significantly mediated relationships between most borderline symptom dimensions and aggression (with negative urgency mediating 7 symptoms and positive urgency 5). The proportion of total effect that was mediated varied between 18.6% (abandonment fears as a predictor of aggression via positive urgency) and 49.8% (quasi psychotic states as a predictor of aggression via negative urgency). Lack of premeditation, albeit to a lesser degree, also mediated the effect of some BPD symptoms on aggression, with a significant mediation effect reported for 4 BPD dimensions, the proportion mediated varying from 7,4 to 20.5%. Sensation seeking significantly mediated the effect of quasi-psychotic symptoms on aggression, with the proportion mediated being 14%. While these results emphasize the role of urgency, particularly negative urgency, in mediating the effects of BPD symptoms on aggression, they also indicate a role for lack of premeditation and sensation seeking. The latter appears to mediate the link between quasi-psychotic symptoms of BPD (paranoid thoughts during times of severe stress) and aggression.

It is notable that in addition to their indirect effects via facets of impulsivity, all borderline symptoms had significant direct effects on aggression. For example, quasi-psychotic symptoms had significant direct effects on aggression in all models shown in Figs. 2, 3 and 4. This strongly suggests that factors other than Urgency may be related to the BPD → aggression link. One other relevant factor is a broad Disagreeableness factor identified by Sleep et al. [47] that reflected functional impairment characterized by aggression, lack of empathy, and interpersonal difficulties. The direct effects on aggression of quasi-psychotic and other symptoms are likely due to their association with this broad Antagonism factor, which – in Sleep et al.’s [47] study – correlated significantly (*r* =.59) with PID-5 Psychoticism. In Bant et al.’s [23] study, PID-5 Psychoticism was significantly associated with both aversively motivated types of aggression (premeditated and controlled), suggesting that psychoticism is associated with aggression in the context of negative, but not positive, affect.

### Study limitations

The study’s main limitation was that it only used self-report measures, which could have introduced bias and made the results seem more significant than they actually were [48]. Another limitation was its use of a short aggression scale, the BAQ-12, that does not sufficiently capture the heterogeneous motivations for aggression. Results suggested it captures primarily impulsive, rather than controlled, aggression, but it does not distinguish between impulsive aggression that is appetitively vs. aversively driven. Future research might attempt to replicate the current results using alternative measures of aggression that capture its motivational heterogeneity.

The cross-sectional nature of the present study, and its lack of prospective data, is another limitation of the present study. Results of the mediation analysis are suggestive only and do not allow inferences regarding temporal causality.

While BPD was the focus of the present study, impulsivity and aggression are transdiagnostic constructs that extend beyond BPD to other syndromes such as ‘malignant narcissism’ [49] and ‘antisocial/borderline comorbidity’ [50]. Indeed, exclusion of borderline PD patients with comorbid Cluster B PDs (antisocial, narcissistic, histrionic and narcissistic) from our borderline PD sample somewhat limits the severity of psychopathology and level of aggression likely to be present (particularly given the “rampant comorbidity” seen in borderline PD [51]). We note that variables other than urgency, for example shame [52], have been shown to mediate the effects of borderline traits on aggression. Indeed, the urgency construct likely embraces impulsive behaviour accompanied by a broad array of emotions, both positive (e.g. excitement) and negative (e.g. shame, anger). Interpersonal stress has also been demonstrated to be a factor that serves to enhance impulsive behavior in the everyday lives of people with personality disorder [53]. A final model of the relationship between BPD and aggression is therefore likely to be considerably more complex than the one shown in Fig. 1. As mentioned above, a general dysfunction factor of Disagreeableness [47] should be included in any complex model, and the role of negative vs. positive affect should be more closely examined.

Finally, due to the nature of our sample, the study could not assess potential gender difference in aggression nor impulsivity, questioning its potential impact on psychopathology [54]. Further study should examine gender difference in these dimension in BPD samples in order to ensure generabilisation of the results.

## Clinical implications

Belligerence, particularly when it presents in the context of negative affect, is a key aspect of the psychopathology seen in patients with borderline symptoms and indicates a need for psychotherapeutic intervention to reduce the risk of aggression. Evidence suggests that the Unified Protocol (UP), a transdiagnostic psychotherapy developed to treat a wide range of emotional disorders characterized by emotion dysregulation [55], can successfully ameliorate symptoms such as excessive shame and dysregulated emotion in adults and adolescents [56–58]. Irritability should arguably be a particular target of UP treatment for borderline patients with high urgency, since the threshold at which irritability transforms into anger appears to be reduced when high levels of urgency are present [59]. Within the framework of the UP, patients could be encouraged to confront, and cope with, their feelings of irritability so that these do not transform into anger. The case of a child with frequent and intense episodes of irritability and anger who was successfully treated using a UP protocol modified for children provides initial support for this approach [60].

## Conclusion

This study provides important information regarding the subtle nature of the relationship between impulsivity, borderline symptoms, and aggression. Results suggest that a close relationship exists between almost all dimensions of BPD, but especially anger, and impulsive aggression. They further suggest that urgency, particularly negative urgency, mediates this relationship. Future studies need to parse aggression into motivationally distinct types.

## Supplementary Information

Supplementary Material 1.

Supplementary Material 2.

## Data Availability

Data will be available under reasonable demand to the corresponding author. The study has not been preregistered.
