# Correction: The relationship between emotional impulsivity (Urgency), aggression, and symptom dimensions in patients with borderline personality disorder

**DOI:** 10.1186/s40479-025-00298-z

**Published:** 2025-05-30

**Authors:** Sylvia Martin, Jonathan Del Monte, Richard Howard

**Affiliations:** 1 Center for Research and Bioethics, Uppsala University, Hursagatan 3, A 11 BMC, Uppsala, Sweden; 2 Psychology Department, Nîmes University, Nîmes, France; 3 Institute of Mental Health, Nottingham, UK

**Correction: Bord Personal Disord Emot Dysregul 12, 19 (2025)**

10.1186/s40479-025-00292-5

Following publication of the original article [1], the authors reported that the name and family name of Sylvia Martin had been mistakenly reversed. The corrected name provided in this Correction is accurate.

The original article [1] has been updated.
