# Supplementary material for: The relationship between emotional impulsivity (Urgency), aggression, and symptom dimensions in patients with borderline personality disorder
Source: Borderline Personal Disord Emot Dysregul. 2025 May 15;12:19. doi: 10.1186/s40479-025-00292-5 (PMC12083163; doi:10.1186/s40479-025-00292-5)
Supplement: Supplementary file 1 — Supplementary Material 1. [file 40479_2025_292_MOESM1_ESM.docx]

Table S1: Means and SD:

|  | Minimum | Maximum | Mean | Std. Deviation |
| --- | --- | --- | --- | --- |
| Time since first diagnosis in months | 0 | 288 | 35.94 | 59.21 |
| age | 16 | 72 | 37.06 | 12.64 |
| Negative Urgency | 4 | 20 | 12.10 | 2.97 |
| Positive Urgency | 4 | 20 | 12.10 | 2.93 |
| Lack of Premeditation | 0 | 16 | 9.19 | 3.14 |
| Lack of Perseverance | 0 | 16 | 8.78 | 3.43 |
| Sensation Seeking | 4 | 20 | 10.49 | 3.59 |
| UPPS | 26 | 77 | 50.98 | 10.25 |
| BAQ12 | 14 | 72 | 40.68 | 12.02 |
| Impulsivity | 0 | 8 | 3.80 | 2.06 |
| Affect Instability | 0 | 10 | 7.68 | 2.45 |
| Abandonment | 0 | 10 | 6.08 | 2.38 |
| Relationships | 0 | 8 | 5.18 | 2.57 |
| Self Image | 0 | 9 | 5.99 | 2.07 |
| Suicide | 0 | 7 | 4.14 | 2.42 |
| Emptiness | 0 | 10 | 7.03 | 2.67 |
| Intense anger | 0 | 10 | 5.75 | 2.82 |
| Quasi Psychotic | 0 | 7 | 2.78 | 2.07 |
| BPQTot | 4 | 74 | 48.45 | 12.99 |
