# Supplementary material for: The relationship between emotional impulsivity (Urgency), aggression, and symptom dimensions in patients with borderline personality disorder
Source: Borderline Personal Disord Emot Dysregul. 2025 May 15;12:19. doi: 10.1186/s40479-025-00292-5 (PMC12083163; doi:10.1186/s40479-025-00292-5)
Supplement: Supplementary file 2 — Supplementary Material 2. [file 40479_2025_292_MOESM2_ESM.docx]

**Impulsivity via Lack of Perseverance to BAQ12**

**Mediation**

| Mediation Estimates | | | | | | | | | | | | | | | | | |
| --- | --- | --- | --- | --- | --- | --- | --- | --- | --- | --- | --- | --- | --- | --- | --- | --- | --- |
|  | | | | | | | | **95% Confidence Interval** | | | |  | | | | | |
| **Effect** | | **Label** | | **Estimate** | | **SE** | | **Lower** | | **Upper** | | **Z** | | **p** | **P’** | **% Mediation** | |
| Indirect |  | a × b |  | -9.80e−4 |  | 0.149 |  | -0.294 |  | 0.292 |  | -0.00656 |  | 0.995 | 4.975 | 0.0452 |  |
| Direct |  | c |  | 2.16 |  | 0.506 |  | 1.173 |  | 3.156 |  | 4.27938 |  | < .001 | <.005 | 99.9548 |  |
| Total |  | c + a × b |  | 2.16 |  | 0.483 |  | 1.216 |  | 3.111 |  | 4.47704 |  | < .001 | **<.005** | 100.0000 |  |
| Note : P´= Bonferroni correction of p, Bonferroni alpha’ = .01 from alpha=0.05 | | | | | | | | | | | | | | | | | |

| Path Estimates | | | | | | | | | | | | | | | | | | | |
| --- | --- | --- | --- | --- | --- | --- | --- | --- | --- | --- | --- | --- | --- | --- | --- | --- | --- | --- | --- |
|  | | | | | | | | | | | | **95% Confidence Interval** | | | |  | | | |
|  | |  | |  | | **Label** | | **Estimate** | | **SE** | | **Lower** | | **Upper** | | **Z** | | **p** | |
| Impulsivity |  | → |  | Lack of Perseverance |  | a |  | 0.51936 |  | 0.146 |  | 0.234 |  | 0.805 |  | 3.56396 |  | < .001 |  |
| Lack of Perseverance |  | → |  | BAQ12 |  | b |  | -0.00189 |  | 0.288 |  | -0.565 |  | 0.562 |  | -0.00656 |  | 0.995 |  |
| Impulsivity |  | → |  | BAQ12 |  | c |  | 2.16442 |  | 0.506 |  | 1.173 |  | 3.156 |  | 4.27938 |  | < .001 |  |
|  | | | | | | | | | | | | | | | | | | | |

Affective instability via Lack of Perseverance to BAQ12

**Mediation**

| Mediation Estimates | | | | | | | | | | | | | | | | | | |
| --- | --- | --- | --- | --- | --- | --- | --- | --- | --- | --- | --- | --- | --- | --- | --- | --- | --- | --- |
|  | | | | | | | | **95% Confidence Interval** | | | |  | | | | | | |
| **Effect** | | **Label** | | **Estimate** | | **SE** | | **Lower** | | **Upper** | | **Z** | | **p** | **P’** | | **% Mediation** | |
| Indirect |  | a × b |  | 0.0300 |  | 0.0445 |  | -0.0572 |  | 0.117 |  | 0.674 |  | 0.500 | | 2.5 | 1.53 |  |
| Direct |  | c |  | 1.9254 |  | 0.3775 |  | 1.1854 |  | 2.665 |  | 5.100 |  | < .001 | | <.005 | 98.47 |  |
| Total |  | c + a × b |  | 1.9554 |  | 0.3771 |  | 1.2162 |  | 2.695 |  | 5.185 |  | < .001 | | **<.005** | 100.00 |  |
| Note : P´= Bonferroni correction of p, Bonferroni alpha’ = .01 from alpha=0.05 | | | | | | | | | | | | | | | | | | |

| Path Estimates | | | | | | | | | | | | | | | | | | | |
| --- | --- | --- | --- | --- | --- | --- | --- | --- | --- | --- | --- | --- | --- | --- | --- | --- | --- | --- | --- |
|  | | | | | | | | | | | | **95% Confidence Interval** | | | |  | | | |
|  | |  | |  | | **Label** | | **Estimate** | | **SE** | | **Lower** | | **Upper** | | **Z** | | **p** | |
| Affect instability |  | → |  | Lack of Perseverance |  | a |  | 0.126 |  | 0.121 |  | -0.112 |  | 0.363 |  | 1.037 |  | 0.300 |  |
| Lack of Perseverance |  | → |  | BAQ12 |  | b |  | 0.239 |  | 0.269 |  | -0.289 |  | 0.766 |  | 0.887 |  | 0.375 |  |
| Affect instability |  | → |  | BAQ12 |  | c |  | 1.925 |  | 0.378 |  | 1.185 |  | 2.665 |  | 5.100 |  | < .001 |  |
|  | | | | | | | | | | | | | | | | | | | |

Abandonment via Lack of Perseverance to BAQ12

**Mediation**

| Mediation Estimates | | | | | | | | | | | | | | | | | | |
| --- | --- | --- | --- | --- | --- | --- | --- | --- | --- | --- | --- | --- | --- | --- | --- | --- | --- | --- |
|  | | | | | | | | **95% Confidence Interval** | | | |  | | | | | | |
| **Effect** | | **Label** | | **Estimate** | | **SE** | | **Lower** | | **Upper** | | **Z** | | **p** | **P’** | | **% Mediation** | |
| Indirect |  | a × b |  | 0.00953 |  | 0.0443 |  | -0.0773 |  | 0.0964 |  | 0.215 |  | 0.830 | | 4.15 | 0.440 |  |
| Direct |  | c |  | 2.15535 |  | 0.4000 |  | 1.3714 |  | 2.9393 |  | 5.389 |  | < .001 | | <.005 | 99.560 |  |
| Total |  | c + a × b |  | 2.16488 |  | 0.4023 |  | 1.3764 |  | 2.9533 |  | 5.381 |  | < .001 | | **<.005** | 100.000 |  |
| Note : P´= Bonferroni correction of p, Bonferroni alpha’ = .01 from alpha=0.05 | | | | | | | | | | | | | | | | | | |

| Path Estimates | | | | | | | | | | | | | | | | | | | |
| --- | --- | --- | --- | --- | --- | --- | --- | --- | --- | --- | --- | --- | --- | --- | --- | --- | --- | --- | --- |
|  | | | | | | | | | | | | **95% Confidence Interval** | | | |  | | | |
|  | |  | |  | | **Label** | | **Estimate** | | **SE** | | **Lower** | | **Upper** | | **Z** | | **p** | |
| Abandonement |  | → |  | Lack of Perseverance |  | a |  | 0.0285 |  | 0.131 |  | -0.227 |  | 0.284 |  | 0.218 |  | 0.827 |  |
| Lack of Perseverance |  | → |  | BAQ12 |  | b |  | 0.3343 |  | 0.266 |  | -0.186 |  | 0.855 |  | 1.259 |  | 0.208 |  |
| Abandonnement |  | → |  | BAQ12 |  | c |  | 2.1553 |  | 0.400 |  | 1.371 |  | 2.939 |  | 5.389 |  | < .001 |  |
|  | | | | | | | | | | | | | | | | | | | |

Relationshipships via Lack of Perseverance to BAQ12

**Mediation**

| Mediation Estimates | | | | | | | | | | | | | | | | | | |
| --- | --- | --- | --- | --- | --- | --- | --- | --- | --- | --- | --- | --- | --- | --- | --- | --- | --- | --- |
|  | | | | | | | | **95% Confidence Interval** | | | |  | | | | | | |
| **Effect** | | **Label** | | **Estimate** | | **SE** | | **Lower** | | **Upper** | | **Z** | | **p** |  | | **% Mediation** | |
| Indirect |  | a × b |  | 0.0246 |  | 0.0424 |  | -0.0585 |  | 0.108 |  | 0.580 |  | 0.562 | | 2.81 | 1.53 |  |
| Direct |  | c |  | 1.5827 |  | 0.3866 |  | 0.8251 |  | 2.340 |  | 4.094 |  | < .001 | | <.005 | 98.47 |  |
| Total |  | c + a × b |  | 1.6073 |  | 0.3875 |  | 0.8478 |  | 2.367 |  | 4.148 |  | < .001 | | **<.005** | 100.00 |  |
| Note : P´= Bonferroni correction of p, Bonferroni alpha’ = .01 from alpha=0.05 | | | | | | | | | | | | | | | | | | |

| Path Estimates | | | | | | | | | | | | | | | | | | | |
| --- | --- | --- | --- | --- | --- | --- | --- | --- | --- | --- | --- | --- | --- | --- | --- | --- | --- | --- | --- |
|  | | | | | | | | | | | | **95% Confidence Interval** | | | |  | | | |
|  | |  | |  | | **Label** | | **Estimate** | | **SE** | | **Lower** | | **Upper** | | **Z** | | **p** | |
| Relationships |  | → |  | Lack of Perseverance |  | a |  | 0.0837 |  | 0.121 |  | -0.153 |  | 0.321 |  | 0.692 |  | 0.489 |  |
| Lack of Perseverance |  | → |  | BAQ12 |  | b |  | 0.2936 |  | 0.277 |  | -0.249 |  | 0.836 |  | 1.061 |  | 0.289 |  |
| Relationships |  | → |  | BAQ12 |  | c |  | 1.5827 |  | 0.387 |  | 0.825 |  | 2.340 |  | 4.094 |  | < .001 |  |
|  | | | | | | | | | | | | | | | | | | | |

Self Image

**Mediation**

| Mediation Estimates | | | | | | | | | | | | | | | | | | |
| --- | --- | --- | --- | --- | --- | --- | --- | --- | --- | --- | --- | --- | --- | --- | --- | --- | --- | --- |
|  | | | | | | | | **95% Confidence Interval** | | | |  | | | | | | |
| **Effect** | | **Label** | | **Estimate** | | **SE** | | **Lower** | | **Upper** | | **Z** | | **p** | **P’** | | **% Mediation** | |
| Indirect |  | a × b |  | 0.0602 |  | 0.0930 |  | -0.122 |  | 0.242 |  | 0.647 |  | 0.518 | | 2.59 | 4.10 |  |
| Direct |  | c |  | 1.4077 |  | 0.4666 |  | 0.493 |  | 2.322 |  | 3.017 |  | 0.003 | | .015 | 95.90 |  |
| Total |  | c + a × b |  | 1.4679 |  | 0.4588 |  | 0.569 |  | 2.367 |  | 3.199 |  | 0.001 | | **.005** | 100.00 |  |
| Note : P´= Bonferroni correction of p, Bonferroni alpha’ = .01 from alpha=0.05 | | | | | | | | | | | | | | | | | | |

| Path Estimates | | | | | | | | | | | | | | | | | | | |
| --- | --- | --- | --- | --- | --- | --- | --- | --- | --- | --- | --- | --- | --- | --- | --- | --- | --- | --- | --- |
|  | | | | | | | | | | | | **95% Confidence Interval** | | | |  | | | |
|  | |  | |  | | **Label** | | **Estimate** | | **SE** | | **Lower** | | **Upper** | | **Z** | | **p** | |
| Self Image |  | → |  | Lack of Perseverance |  | a |  | 0.308 |  | 0.138 |  | 0.0387 |  | 0.578 |  | 2.241 |  | 0.025 |  |
| Lack of Perseverance |  | → |  | BAQ12 |  | b |  | 0.195 |  | 0.289 |  | -0.3708 |  | 0.761 |  | 0.676 |  | 0.499 |  |
| Self Image |  | → |  | BAQ12 |  | c |  | 1.408 |  | 0.467 |  | 0.4931 |  | 2.322 |  | 3.017 |  | 0.003 |  |
|  | | | | | | | | | | | | | | | | | | | |

Suicide

**Mediation**

| Mediation Estimates | | | | | | | | | | | | | | | | | | |
| --- | --- | --- | --- | --- | --- | --- | --- | --- | --- | --- | --- | --- | --- | --- | --- | --- | --- | --- |
|  | | | | | | | | **95% Confidence Interval** | | | |  | | | | | | |
| **Effect** | | **Label** | | **Estimate** | | **SE** | | **Lower** | | **Upper** | | **Z** | | **p** | **P’** | | **% Mediation** | |
| Indirect |  | a × b |  | -0.240 |  | 0.155 |  | -0.544 |  | 0.0647 |  | -1.544 |  | 0.123 | | .615 | 27.2 |  |
| Direct |  | c |  | 0.643 |  | 0.432 |  | -0.204 |  | 1.4897 |  | 1.489 |  | 0.137 | | .685 | 72.8 |  |
| Total |  | c + a × b |  | 0.403 |  | 0.412 |  | -0.404 |  | 1.2098 |  | 0.979 |  | 0.327 | | 1.635 | 100.0 |  |
| Note : P´= Bonferroni correction of p, Bonferroni alpha’ = .01 from alpha=0.05 | | | | | | | | | | | | | | | | | | |

| Path Estimates | | | | | | | | | | | | | | | | | | | |
| --- | --- | --- | --- | --- | --- | --- | --- | --- | --- | --- | --- | --- | --- | --- | --- | --- | --- | --- | --- |
|  | | | | | | | | | | | | **95% Confidence Interval** | | | |  | | | |
|  | |  | |  | | **Label** | | **Estimate** | | **SE** | | **Lower** | | **Upper** | | **Z** | | **p** | |
| Suicide |  | → |  | Lack of Perseverance |  | a |  | -0.467 |  | 0.115 |  | -0.6912 |  | -0.242 |  | -4.07 |  | < .001 |  |
| Lack of Perseverance |  | → |  | BAQ12 |  | b |  | 0.514 |  | 0.308 |  | -0.0899 |  | 1.118 |  | 1.67 |  | 0.095 |  |
| Suicide |  | → |  | BAQ12 |  | c |  | 0.643 |  | 0.432 |  | -0.2036 |  | 1.490 |  | 1.49 |  | 0.137 |  |
|  | | | | | | | | | | | | | | | | | | | |

Emptiness

**Mediation**

| Mediation Estimates | | | | | | | | | | | | | | | | | | |
| --- | --- | --- | --- | --- | --- | --- | --- | --- | --- | --- | --- | --- | --- | --- | --- | --- | --- | --- |
|  | | | | | | | | **95% Confidence Interval** | | | |  | | | | | | |
| **Effect** | | **Label** | | **Estimate** | | **SE** | | **Lower** | | **Upper** | | **Z** | | **p** | **P’** | | **% Mediation** | |
| Indirect |  | a × b |  | 0.00218 |  | 0.0861 |  | -0.167 |  | 0.171 |  | 0.0253 |  | 0.980 | | 4.9 | 0.113 |  |
| Direct |  | c |  | 1.92442 |  | 0.3577 |  | 1.223 |  | 2.625 |  | 5.3803 |  | < .001 | | <.005 | 99.887 |  |
| Total |  | c + a × b |  | 1.92660 |  | 0.3472 |  | 1.246 |  | 2.607 |  | 5.5497 |  | < .001 | | **<.005** | 100.000 |  |
| Note : P´= Bonferroni correction of p, Bonferroni alpha’ = .01 from alpha=0.05 | | | | | | | | | | | | | | | | | | |

| Path Estimates | | | | | | | | | | | | | | | | | | | |
| --- | --- | --- | --- | --- | --- | --- | --- | --- | --- | --- | --- | --- | --- | --- | --- | --- | --- | --- | --- |
|  | | | | | | | | | | | | **95% Confidence Interval** | | | |  | | | |
|  | |  | |  | | **Label** | | **Estimate** | | **SE** | | **Lower** | | **Upper** | | **Z** | | **p** | |
| Emptiness |  | → |  | Lack of Perseverance |  | a |  | 0.31483 |  | 0.110 |  | 0.0992 |  | 0.530 |  | 2.8615 |  | 0.004 |  |
| Lack of Perseverance |  | → |  | BAQ12 |  | b |  | 0.00693 |  | 0.274 |  | -0.5293 |  | 0.543 |  | 0.0253 |  | 0.980 |  |
| Emptiness |  | → |  | BAQ12 |  | c |  | 1.92442 |  | 0.358 |  | 1.2234 |  | 2.625 |  | 5.3803 |  | < .001 |  |
|  | | | | | | | | | | | | | | | | | | | |

Intense Anger

**Mediation**

| Mediation Estimates | | | | | | | | | | | | | | | | | | |
| --- | --- | --- | --- | --- | --- | --- | --- | --- | --- | --- | --- | --- | --- | --- | --- | --- | --- | --- |
|  | | | | | | | | **95% Confidence Interval** | | | |  | | | | | | |
| **Effect** | | **Label** | | **Estimate** | | **SE** | | **Lower** | | **Upper** | | **Z** | | **p** | **P’** | | **% Mediation** | |
| Indirect |  | a × b |  | 0.0140 |  | 0.0379 |  | -0.0602 |  | 0.0882 |  | 0.369 |  | 0.712 | | 3.56 | 0.525 |  |
| Direct |  | c |  | 2.6515 |  | 0.2861 |  | 2.0907 |  | 3.2123 |  | 9.267 |  | < .001 | | <.005 | 99.475 |  |
| Total |  | c + a × b |  | 2.6655 |  | 0.2839 |  | 2.1090 |  | 3.2220 |  | 9.388 |  | < .001 | | **<.005** | 100.000 |  |
| Note : P´= Bonferroni correction of p, Bonferroni alpha’ = .01 from alpha=0.05 | | | | | | | | | | | | | | | | | | |

| Path Estimates | | | | | | | | | | | | | | | | | | | |
| --- | --- | --- | --- | --- | --- | --- | --- | --- | --- | --- | --- | --- | --- | --- | --- | --- | --- | --- | --- |
|  | | | | | | | | | | | | **95% Confidence Interval** | | | |  | | | |
|  | |  | |  | | **Label** | | **Estimate** | | **SE** | | **Lower** | | **Upper** | | **Z** | | **p** | |
| Intense Anger |  | → |  | Lack of Perseverance |  | a |  | 0.1593 |  | 0.107 |  | -0.0501 |  | 0.369 |  | 1.491 |  | 0.136 |  |
| Lack of Perseverance |  | → |  | BAQ12 |  | b |  | 0.0878 |  | 0.230 |  | -0.3636 |  | 0.539 |  | 0.381 |  | 0.703 |  |
| Intense Anger |  | → |  | BAQ12 |  | c |  | 2.6515 |  | 0.286 |  | 2.0907 |  | 3.212 |  | 9.267 |  | < .001 |  |
| Note : P´= Bonferroni correction of p, Bonferroni alpha’ = .01 from alpha=0.05 | | | | | | | | | | | | | | | | | | | |

Psychotic

**Mediation**

| Mediation Estimates | | | | | | | | | | | | | | | | | | |
| --- | --- | --- | --- | --- | --- | --- | --- | --- | --- | --- | --- | --- | --- | --- | --- | --- | --- | --- |
|  | | | | | | | | **95% Confidence Interval** | | | |  | | | | | | |
| **Effect** | | **Label** | | **Estimate** | | **SE** | | **Lower** | | **Upper** | | **Z** | | **p** | **P’** | | **% Mediation** | |
| Indirect |  | a × b |  | -0.0187 |  | 0.0622 |  | -0.141 |  | 0.103 |  | -0.301 |  | 0.763 | | 3.815 | 0.921 |  |
| Direct |  | c |  | 2.0144 |  | 0.4949 |  | 1.044 |  | 2.984 |  | 4.071 |  | < .001 | | <.005 | 99.079 |  |
| Total |  | c + a × b |  | 1.9956 |  | 0.4984 |  | 1.019 |  | 2.972 |  | 4.004 |  | < .001 | | **<.005** | 100.000 |  |
| Note : P´= Bonferroni correction of p, Bonferroni alpha’ = .01 from alpha=0.05 | | | | | | | | | | | | | | | | | | |

| Path Estimates | | | | | | | | | | | | | | | | | | | |
| --- | --- | --- | --- | --- | --- | --- | --- | --- | --- | --- | --- | --- | --- | --- | --- | --- | --- | --- | --- |
|  | | | | | | | | | | | | **95% Confidence Interval** | | | |  | | | |
|  | |  | |  | | **Label** | | **Estimate** | | **SE** | | **Lower** | | **Upper** | | **Z** | | **p** | |
| Quasi Psychotic |  | → |  | Lack of Perseverance |  | a |  | -0.0478 |  | 0.155 |  | -0.352 |  | 0.256 |  | -0.308 |  | 0.758 |  |
| Lack of Perseverance |  | → |  | BAQ12 |  | b |  | 0.3915 |  | 0.276 |  | -0.150 |  | 0.933 |  | 1.416 |  | 0.157 |  |
| Quasi Psychotic |  | → |  | BAQ12 |  | c |  | 2.0144 |  | 0.495 |  | 1.044 |  | 2.984 |  | 4.071 |  | < .001 |  |
|  | | | | | | | | | | | | | | | | | | | |

**Impulsivity via Lack of Premeditation to BAQ12**

**Mediation**

| Mediation Estimates | | | | | | | | | | | | | | | | | | |
| --- | --- | --- | --- | --- | --- | --- | --- | --- | --- | --- | --- | --- | --- | --- | --- | --- | --- | --- |
|  | | | | | | | | **95% Confidence Interval** | | | |  | | | | | | |
| **Effect** | | **Label** | | **Estimate** | | **SE** | | **Lower** | | **Upper** | | **Z** | | **p** | **P’** | | **% Mediation** | |
| Indirect |  | a × b |  | 0.314 |  | 0.237 |  | -0.150 |  | 0.777 |  | 1.33 |  | 0.185 | | .925 | 14.5 |  |
| Direct |  | c |  | 1.850 |  | 0.532 |  | 0.807 |  | 2.892 |  | 3.48 |  | < .001 | | <.005 | 85.5 |  |
| Total |  | c + a × b |  | 2.163 |  | 0.483 |  | 1.216 |  | 3.111 |  | 4.48 |  | < .001 | | **<.005** | 100.0 |  |
| Note : P´= Bonferroni correction of p, Bonferroni alpha’ = .01 from alpha=0.05 | | | | | | | | | | | | | | | | | | |

| Path Estimates | | | | | | | | | | | | | | | | | | | |
| --- | --- | --- | --- | --- | --- | --- | --- | --- | --- | --- | --- | --- | --- | --- | --- | --- | --- | --- | --- |
|  | | | | | | | | | | | | **95% Confidence Interval** | | | |  | | | |
|  | |  | |  | | **Label** | | **Estimate** | | **SE** | | **Lower** | | **Upper** | | **Z** | | **p** | |
| Impulsivity |  | → |  | Lack of Premeditation |  | a |  | 0.666 |  | 0.121 |  | 0.429 |  | 0.903 |  | 5.52 |  | < .001 |  |
| Lack of Premeditation |  | → |  | BAQ12 |  | b |  | 0.471 |  | 0.345 |  | -0.205 |  | 1.147 |  | 1.37 |  | 0.172 |  |
| Impulsivity |  | → |  | BAQ12 |  | c |  | 1.850 |  | 0.532 |  | 0.807 |  | 2.892 |  | 3.48 |  | < .001 |  |
|  | | | | | | | | | | | | | | | | | | | |

Affect instability via Lack of Premeditation to BAQ12

**Mediation**

| Mediation Estimates | | | | | | | | | | | | | | | | | | |
| --- | --- | --- | --- | --- | --- | --- | --- | --- | --- | --- | --- | --- | --- | --- | --- | --- | --- | --- |
|  | | | | | | | | **95% Confidence Interval** | | | |  | | | | | | |
| **Effect** | | **Label** | | **Estimate** | | **SE** | | **Lower** | | **Upper** | | **Z** | | **p** | **P’** | | **% Mediation** | |
| Indirect |  | a × b |  | 0.212 |  | 0.147 |  | -0.0756 |  | 0.500 |  | 1.45 |  | 0.148 | | .74 | 10.9 |  |
| Direct |  | c |  | 1.743 |  | 0.399 |  | 0.9620 |  | 2.524 |  | 4.37 |  | < .001 | | <.005 | 89.1 |  |
| Total |  | c + a × b |  | 1.955 |  | 0.377 |  | 1.2162 |  | 2.695 |  | 5.18 |  | < .001 | | **<.005** | 100.0 |  |
| Note : P´= Bonferroni correction of p, Bonferroni alpha’ = .01 from alpha=0.05 | | | | | | | | | | | | | | | | | | |

| Path Estimates | | | | | | | | | | | | | | | | | | | |
| --- | --- | --- | --- | --- | --- | --- | --- | --- | --- | --- | --- | --- | --- | --- | --- | --- | --- | --- | --- |
|  | | | | | | | | | | | | **95% Confidence Interval** | | | |  | | | |
|  | |  | |  | | **Label** | | **Estimate** | | **SE** | | **Lower** | | **Upper** | | **Z** | | **p** | |
| Affect instability |  | → |  | Lack of Premeditation |  | a |  | 0.427 |  | 0.100 |  | 0.230 |  | 0.623 |  | 4.26 |  | < .001 |  |
| Lack of Premeditation |  | → |  | BAQ12 |  | b |  | 0.497 |  | 0.324 |  | -0.137 |  | 1.132 |  | 1.54 |  | 0.124 |  |
| Affect instability |  | → |  | BAQ12 |  | c |  | 1.743 |  | 0.399 |  | 0.962 |  | 2.524 |  | 4.37 |  | < .001 |  |
|  | | | | | | | | | | | | | | | | | | | |

Abandonment via Lack of Premeditation to BAQ12

**Mediation**

| Mediation Estimates | | | | | | | | | | | | | | | | | | |
| --- | --- | --- | --- | --- | --- | --- | --- | --- | --- | --- | --- | --- | --- | --- | --- | --- | --- | --- |
|  | | | | | | | | **95% Confidence Interval** | | | |  | | | | | | |
| **Effect** | | **Label** | | **Estimate** | | **SE** | | **Lower** | | **Upper** | | **Z** | | **p** | **P’** | | **% Mediation** | |
| Indirect |  | a × b |  | 0.160 |  | 0.106 |  | -0.0478 |  | 0.368 |  | 1.51 |  | 0.131 | | .655 | 7.40 |  |
| Direct |  | c |  | 2.005 |  | 0.399 |  | 1.2228 |  | 2.786 |  | 5.03 |  | < .001 | | <.005 | 92.60 |  |
| Total |  | c + a × b |  | 2.165 |  | 0.402 |  | 1.3764 |  | 2.953 |  | 5.38 |  | < .001 | | **<.005** | 100.00 |  |
| Note : P´= Bonferroni correction of p, Bonferroni alpha’ = .01 from alpha=0.05 | | | | | | | | | | | | | | | | | | |

| Path Estimates | | | | | | | | | | | | | | | | | | | |
| --- | --- | --- | --- | --- | --- | --- | --- | --- | --- | --- | --- | --- | --- | --- | --- | --- | --- | --- | --- |
|  | | | | | | | | | | | | **95% Confidence Interval** | | | |  | | | |
|  | |  | |  | | **Label** | | **Estimate** | | **SE** | | **Lower** | | **Upper** | | **Z** | | **p** | |
| Abandonnement |  | → |  | Lack of Premeditation |  | a |  | 0.217 |  | 0.113 |  | -0.00482 |  | 0.438 |  | 1.92 |  | 0.055 |  |
| Lack of Premeditation |  | → |  | BAQ12 |  | b |  | 0.740 |  | 0.302 |  | 0.14789 |  | 1.331 |  | 2.45 |  | 0.014 |  |
| Abandonnement |  | → |  | BAQ12 |  | c |  | 2.005 |  | 0.399 |  | 1.22276 |  | 2.786 |  | 5.03 |  | < .001 |  |
|  | | | | | | | | | | | | | | | | | | | |

Relationships via Lack of Premeditation to BAQ12

**Mediation**

| Mediation Estimates | | | | | | | | | | | | | | | | | | |
| --- | --- | --- | --- | --- | --- | --- | --- | --- | --- | --- | --- | --- | --- | --- | --- | --- | --- | --- |
|  | | | | | | | | **95% Confidence Interval** | | | |  | | | | | | |
| **Effect** | | **Label** | | **Estimate** | | **SE** | | **Lower** | | **Upper** | | **Z** | | **p** | **P’** | | **% Mediation** | |
| Indirect |  | a × b |  | 0.187 |  | 0.111 |  | -0.0303 |  | 0.405 |  | 1.69 |  | 0.092 | | .46 | 11.7 |  |
| Direct |  | c |  | 1.420 |  | 0.388 |  | 0.6603 |  | 2.180 |  | 3.66 |  | < .001 | | <.005 | 88.3 |  |
| Total |  | c + a × b |  | 1.607 |  | 0.388 |  | 0.8478 |  | 2.367 |  | 4.15 |  | < .001 | | **<.005** | 100.0 |  |
| Note : P´= Bonferroni correction of p, Bonferroni alpha’ = .01 from alpha=0.05 | | | | | | | | | | | | | | | | | | |

| Path Estimates | | | | | | | | | | | | | | | | | | | |
| --- | --- | --- | --- | --- | --- | --- | --- | --- | --- | --- | --- | --- | --- | --- | --- | --- | --- | --- | --- |
|  | | | | | | | | | | | | **95% Confidence Interval** | | | |  | | | |
|  | |  | |  | | **Label** | | **Estimate** | | **SE** | | **Lower** | | **Upper** | | **Z** | | **p** | |
| Relationships |  | → |  | Lack of Premeditation |  | a |  | 0.249 |  | 0.104 |  | 0.0448 |  | 0.453 |  | 2.39 |  | 0.017 |  |
| Lack of Premeditation |  | → |  | BAQ12 |  | b |  | 0.753 |  | 0.316 |  | 0.1336 |  | 1.373 |  | 2.38 |  | 0.017 |  |
| Relationships |  | → |  | BAQ12 |  | c |  | 1.420 |  | 0.388 |  | 0.6603 |  | 2.180 |  | 3.66 |  | < .001 |  |
|  | | | | | | | | | | | | | | | | | | | |

Self-Image via Lack of Premeditation to BAQ12

**Mediation**

| Mediation Estimates | | | | | | | | | | | | | | | | | | |
| --- | --- | --- | --- | --- | --- | --- | --- | --- | --- | --- | --- | --- | --- | --- | --- | --- | --- | --- |
|  | | | | | | | | **95% Confidence Interval** | | | |  | | | | | | |
| **Effect** | | **Label** | | **Estimate** | | **SE** | | **Lower** | | **Upper** | | **Z** | | **p** | **P’** | | **% Mediation** | |
| Indirect |  | a × b |  | 0.300 |  | 0.159 |  | -0.0106 |  | 0.611 |  | 1.89 |  | 0.058 | | .29 | 20.5 |  |
| Direct |  | c |  | 1.168 |  | 0.469 |  | 0.2489 |  | 2.086 |  | 2.49 |  | 0.013 | | .065 | 79.5 |  |
| Total |  | c + a × b |  | 1.468 |  | 0.459 |  | 0.5686 |  | 2.367 |  | 3.20 |  | 0.001 | | **.005** | 100.0 |  |
| Note : P´= Bonferroni correction of p, Bonferroni alpha’ = .01 from alpha=0.05 | | | | | | | | | | | | | | | | | | |

| Path Estimates | | | | | | | | | | | | | | | | | | | |
| --- | --- | --- | --- | --- | --- | --- | --- | --- | --- | --- | --- | --- | --- | --- | --- | --- | --- | --- | --- |
|  | | | | | | | | | | | | **95% Confidence Interval** | | | |  | | | |
|  | |  | |  | | **Label** | | **Estimate** | | **SE** | | **Lower** | | **Upper** | | **Z** | | **p** | |
| Self Image |  | → |  | Lack of Premeditation |  | a |  | 0.396 |  | 0.118 |  | 0.165 |  | 0.627 |  | 3.36 |  | < .001 |  |
| Lack of Premeditation |  | → |  | BAQ12 |  | b |  | 0.758 |  | 0.331 |  | 0.110 |  | 1.406 |  | 2.29 |  | 0.022 |  |
| Self Image |  | → |  | BAQ12 |  | c |  | 1.168 |  | 0.469 |  | 0.249 |  | 2.086 |  | 2.49 |  | 0.013 |  |
|  | | | | | | | | | | | | | | | | | | | |

Suicide via Lack of Premeditation to BAQ12

**Mediation**

| Mediation Estimates | | | | | | | | | | | | | | | | | | |
| --- | --- | --- | --- | --- | --- | --- | --- | --- | --- | --- | --- | --- | --- | --- | --- | --- | --- | --- |
|  | | | | | | | | **95% Confidence Interval** | | | |  | | | | | | |
| **Effect** | | **Label** | | **Estimate** | | **SE** | | **Lower** | | **Upper** | | **Z** | | **p** | **P’** | | **% Mediation** | |
| Indirect |  | a × b |  | -0.229 |  | 0.133 |  | -0.489 |  | 0.0312 |  | -1.725 |  | 0.085 | | .425 | 26.6 |  |
| Direct |  | c |  | 0.632 |  | 0.402 |  | -0.155 |  | 1.4197 |  | 1.573 |  | 0.116 | | .58 | 73.4 |  |
| Total |  | c + a × b |  | 0.403 |  | 0.412 |  | -0.404 |  | 1.2098 |  | 0.979 |  | 0.327 | | 1.635 | 100.0 |  |
| Note : P´= Bonferroni correction of p, Bonferroni alpha’ = .01 from alpha=0.05 | | | | | | | | | | | | | | | | | | |

| Path Estimates | | | | | | | | | | | | | | | | | | | |
| --- | --- | --- | --- | --- | --- | --- | --- | --- | --- | --- | --- | --- | --- | --- | --- | --- | --- | --- | --- |
|  | | | | | | | | | | | | **95% Confidence Interval** | | | |  | | | |
|  | |  | |  | | **Label** | | **Estimate** | | **SE** | | **Lower** | | **Upper** | | **Z** | | **p** | |
| Suicide |  | → |  | Lack of Premeditation |  | a |  | -0.213 |  | 0.105 |  | -0.418 |  | -0.00677 |  | -2.02 |  | 0.043 |  |
| Lack of Premeditation |  | → |  | BAQ12 |  | b |  | 1.077 |  | 0.327 |  | 0.437 |  | 1.71762 |  | 3.30 |  | < .001 |  |
| Suicide |  | → |  | BAQ12 |  | c |  | 0.632 |  | 0.402 |  | -0.155 |  | 1.41966 |  | 1.57 |  | 0.116 |  |
|  | | | | | | | | | | | | | | | | | | | |

Intense Anger via Lack of Premeditation to BAQ12

**Mediation**

| Mediation Estimates | | | | | | | | | | | | | | | | | | |
| --- | --- | --- | --- | --- | --- | --- | --- | --- | --- | --- | --- | --- | --- | --- | --- | --- | --- | --- |
|  | | | | | | | | **95% Confidence Interval** | | | |  | | | | | | |
| **Effect** | | **Label** | | **Estimate** | | **SE** | | **Lower** | | **Upper** | | **Z** | | **p** | **P’** | | **% Mediation** | |
| Indirect |  | a × b |  | 0.168 |  | 0.102 |  | -0.0314 |  | 0.368 |  | 1.65 |  | 0.099 | | .495 | 8.73 |  |
| Direct |  | c |  | 1.758 |  | 0.351 |  | 1.0699 |  | 2.447 |  | 5.01 |  | < .001 | | <.005 | 91.27 |  |
| Total |  | c + a × b |  | 1.927 |  | 0.347 |  | 1.2462 |  | 2.607 |  | 5.55 |  | < .001 | | **<.005** | 100.00 |  |
| Note : P´= Bonferroni correction of p, Bonferroni alpha’ = .01 from alpha=0.05 | | | | | | | | | | | | | | | | | | |

| Path Estimates | | | | | | | | | | | | | | | | | | | |
| --- | --- | --- | --- | --- | --- | --- | --- | --- | --- | --- | --- | --- | --- | --- | --- | --- | --- | --- | --- |
|  | | | | | | | | | | | | **95% Confidence Interval** | | | |  | | | |
|  | |  | |  | | **Label** | | **Estimate** | | **SE** | | **Lower** | | **Upper** | | **Z** | | **p** | |
| Emptiness |  | → |  | Lack of Premeditation |  | a |  | 0.266 |  | 0.0967 |  | 0.0762 |  | 0.455 |  | 2.75 |  | 0.006 |  |
| Lack of Premeditation |  | → |  | BAQ12 |  | b |  | 0.633 |  | 0.3063 |  | 0.0327 |  | 1.233 |  | 2.07 |  | 0.039 |  |
| Emptiness |  | → |  | BAQ12 |  | c |  | 1.758 |  | 0.3513 |  | 1.0699 |  | 2.447 |  | 5.01 |  | < .001 |  |
|  | | | | | | | | | | | | | | | | | | | |

Psychotic via Lack of Premeditation to BAQ12

**Mediation**

| Mediation Estimates | | | | | | | | | | | | | | | | | | |
| --- | --- | --- | --- | --- | --- | --- | --- | --- | --- | --- | --- | --- | --- | --- | --- | --- | --- | --- |
|  | | | | | | | | **95% Confidence Interval** | | | |  | | | | | | |
| **Effect** | | **Label** | | **Estimate** | | **SE** | | **Lower** | | **Upper** | | **Z** | | **p** | **P’** | | **% Mediation** | |
| Indirect |  | a × b |  | 0.0279 |  | 0.132 |  | -0.230 |  | 0.286 |  | 0.212 |  | 0.832 | | 4.16 | 1.40 |  |
| Direct |  | c |  | 1.9677 |  | 0.481 |  | 1.025 |  | 2.910 |  | 4.092 |  | < .001 | | <.005 | 98.60 |  |
| Total |  | c + a × b |  | 1.9956 |  | 0.498 |  | 1.019 |  | 2.972 |  | 4.004 |  | < .001 | | **<.005** | 100.00 |  |
| Note : P´= Bonferroni correction of p, Bonferroni alpha’ = .01 from alpha=0.05 | | | | | | | | | | | | | | | | | | |

| Path Estimates | | | | | | | | | | | | | | | | | | | |
| --- | --- | --- | --- | --- | --- | --- | --- | --- | --- | --- | --- | --- | --- | --- | --- | --- | --- | --- | --- |
|  | | | | | | | | | | | | **95% Confidence Interval** | | | |  | | | |
|  | |  | |  | | **Label** | | **Estimate** | | **SE** | | **Lower** | | **Upper** | | **Z** | | **p** | |
| Quasi Psychotic |  | → |  | Lack of Premeditation |  | a |  | 0.0289 |  | 0.136 |  | -0.238 |  | 0.296 |  | 0.212 |  | 0.832 |  |
| Lack of Premeditation |  | → |  | BAQ12 |  | b |  | 0.9652 |  | 0.306 |  | 0.365 |  | 1.565 |  | 3.153 |  | 0.002 |  |
| Quasi Psychotic |  | → |  | BAQ12 |  | c |  | 1.9677 |  | 0.481 |  | 1.025 |  | 2.910 |  | 4.092 |  | < .001 |  |
|  | | | | | | | | | | | | | | | | | | | |

**Impulsivity via Negative urgency to BAQ12**

**Mediation**

| Mediation Estimates | | | | | | | | | | | | | | | | | | |
| --- | --- | --- | --- | --- | --- | --- | --- | --- | --- | --- | --- | --- | --- | --- | --- | --- | --- | --- |
|  | | | | | | | | **95% Confidence Interval** | | | |  | | | | | | |
| **Effect** | | **Label** | | **Estimate** | | **SE** | | **Lower** | | **Upper** | | **Z** | | **p** | **P’** | | **% Mediation** | |
| Indirect |  | a × b |  | 1.02 |  | 0.286 |  | 0.460 |  | 1.58 |  | 3.57 |  | < .001 | | <.005 | 47.2 |  |
| Direct |  | c |  | 1.14 |  | 0.446 |  | 0.269 |  | 2.02 |  | 2.56 |  | 0.010 | | 0.050 | 52.8 |  |
| Total |  | c + a × b |  | 2.16 |  | 0.483 |  | 1.216 |  | 3.11 |  | 4.48 |  | < .001 | | **<.005** | 100.0 |  |
| Note : P´= Bonferroni correction of p, Bonferroni alpha’ = .01 from alpha=0.05 | | | | | | | | | | | | | | | | | | |

| Path Estimates | | | | | | | | | | | | | | | | | | | |
| --- | --- | --- | --- | --- | --- | --- | --- | --- | --- | --- | --- | --- | --- | --- | --- | --- | --- | --- | --- |
|  | | | | | | | | | | | | **95% Confidence Interval** | | | |  | | | |
|  | |  | |  | | **Label** | | **Estimate** | | **SE** | | **Lower** | | **Upper** | | **Z** | | **p** | |
| Impulsivity |  | → |  | Negative Urgency |  | a |  | 0.488 |  | 0.115 |  | 0.262 |  | 0.714 |  | 4.23 |  | < .001 |  |
| Negative Urgency |  | → |  | BAQ12 |  | b |  | 2.092 |  | 0.315 |  | 1.475 |  | 2.709 |  | 6.64 |  | < .001 |  |
| Impulsivity |  | → |  | BAQ12 |  | c |  | 1.143 |  | 0.446 |  | 0.269 |  | 2.017 |  | 2.56 |  | 0.010 |  |
|  | | | | | | | | | | | | | | | | | | | |

Affective instability via Negative urgency to BAQ12

**Mediation**

| Mediation Estimates | | | | | | | | | | | | | | | | | | |
| --- | --- | --- | --- | --- | --- | --- | --- | --- | --- | --- | --- | --- | --- | --- | --- | --- | --- | --- |
|  | | | | | | | | **95% Confidence Interval** | | | |  | | | | | | |
| **Effect** | | **Label** | | **Estimate** | | **SE** | | **Lower** | | **Upper** | | **Z** | | **p** | **P’** | | **% Mediation** | |
| Indirect |  | a × b |  | 0.801 |  | 0.222 |  | 0.366 |  | 1.24 |  | 3.61 |  | < .001 | | <.005 | 41.0 |  |
| Direct |  | c |  | 1.155 |  | 0.352 |  | 0.465 |  | 1.84 |  | 3.28 |  | 0.001 | | .005 | 59.0 |  |
| Total |  | c + a × b |  | 1.955 |  | 0.377 |  | 1.216 |  | 2.69 |  | 5.18 |  | < .001 | | **<.005** | 100.0 |  |
| Note : P´= Bonferroni correction of p, Bonferroni alpha’ = .01 from alpha=0.05 | | | | | | | | | | | | | | | | | | |

| Path Estimates | | | | | | | | | | | | | | | | | | | |
| --- | --- | --- | --- | --- | --- | --- | --- | --- | --- | --- | --- | --- | --- | --- | --- | --- | --- | --- | --- |
|  | | | | | | | | | | | | **95% Confidence Interval** | | | |  | | | |
|  | |  | |  | | **Label** | | **Estimate** | | **SE** | | **Lower** | | **Upper** | | **Z** | | **p** | |
| Affect instability |  | → |  | Negative Urgency |  | a |  | 0.398 |  | 0.0917 |  | 0.219 |  | 0.578 |  | 4.35 |  | < .001 |  |
| Negative Urgency |  | → |  | BAQ12 |  | b |  | 2.010 |  | 0.3114 |  | 1.399 |  | 2.620 |  | 6.45 |  | < .001 |  |
| Affect instability |  | → |  | BAQ12 |  | c |  | 1.155 |  | 0.3517 |  | 0.465 |  | 1.844 |  | 3.28 |  | 0.001 |  |
|  | | | | | | | | | | | | | | | | | | | |

Abandonment via Negative urgency to BAQ12

**Mediation**

| Mediation Estimates | | | | | | | | | | | | | | | | | | |
| --- | --- | --- | --- | --- | --- | --- | --- | --- | --- | --- | --- | --- | --- | --- | --- | --- | --- | --- |
|  | | | | | | | | **95% Confidence Interval** | | | |  | | | | | | |
| **Effect** | | **Label** | | **Estimate** | | **SE** | | **Lower** | | **Upper** | | **Z** | | **p** | **P’** | | **% Mediation** | |
| Indirect |  | a × b |  | 1.04 |  | 0.254 |  | 0.544 |  | 1.54 |  | 4.10 |  | < .001 | | <.005 | 48.2 |  |
| Direct |  | c |  | 1.12 |  | 0.397 |  | 0.345 |  | 1.90 |  | 2.83 |  | 0.005 | | .025 | 51.8 |  |
| Total |  | c + a × b |  | 2.16 |  | 0.402 |  | 1.376 |  | 2.95 |  | 5.38 |  | < .001 | | **<.005** | 100.0 |  |
| Note : P´= Bonferroni correction of p, Bonferroni alpha’ = .01 from alpha=0.05 | | | | | | | | | | | | | | | | | | |

| Path Estimates | | | | | | | | | | | | | | | | | | | |
| --- | --- | --- | --- | --- | --- | --- | --- | --- | --- | --- | --- | --- | --- | --- | --- | --- | --- | --- | --- |
|  | | | | | | | | | | | | **95% Confidence Interval** | | | |  | | | |
|  | |  | |  | | **Label** | | **Estimate** | | **SE** | | **Lower** | | **Upper** | | **Z** | | **p** | |
| Abandonnement |  | → |  | Negative Urgency |  | a |  | 0.530 |  | 0.0946 |  | 0.345 |  | 0.716 |  | 5.61 |  | < .001 |  |
| Negative Urgency |  | → |  | BAQ12 |  | b |  | 1.965 |  | 0.3272 |  | 1.324 |  | 2.607 |  | 6.01 |  | < .001 |  |
| Abandonnement |  | → |  | BAQ12 |  | c |  | 1.122 |  | 0.3968 |  | 0.345 |  | 1.900 |  | 2.83 |  | 0.005 |  |
|  | | | | | | | | | | | | | | | | | | | |

Relationships via Negative urgency to BAQ12

**Mediation**

| Mediation Estimates | | | | | | | | | | | | | | | | | | |
| --- | --- | --- | --- | --- | --- | --- | --- | --- | --- | --- | --- | --- | --- | --- | --- | --- | --- | --- |
|  | | | | | | | | **95% Confidence Interval** | | | |  | | | | | | |
| **Effect** | | **Label** | | **Estimate** | | **SE** | | **Lower** | | **Upper** | | **Z** | | **p** | **P’** | | **% Mediation** | |
| Indirect |  | a × b |  | 1.090 |  | 0.254 |  | 0.593 |  | 1.59 |  | 4.30 |  | < .001 | | <.005 | 67.8 |  |
| Direct |  | c |  | 0.518 |  | 0.378 |  | -0.224 |  | 1.26 |  | 1.37 |  | 0.171 | | .855 | 32.2 |  |
| Total |  | c + a × b |  | 1.607 |  | 0.388 |  | 0.848 |  | 2.37 |  | 4.15 |  | < .001 | | **<.005** | 100.0 |  |
| Note : P´= Bonferroni correction of p, Bonferroni alpha’ = .01 from alpha=0.05 | | | | | | | | | | | | | | | | | | |

| Path Estimates | | | | | | | | | | | | | | | | | | | |
| --- | --- | --- | --- | --- | --- | --- | --- | --- | --- | --- | --- | --- | --- | --- | --- | --- | --- | --- | --- |
|  | | | | | | | | | | | | **95% Confidence Interval** | | | |  | | | |
|  | |  | |  | | **Label** | | **Estimate** | | **SE** | | **Lower** | | **Upper** | | **Z** | | **p** | |
| Relationships |  | → |  | Negative Urgency |  | a |  | 0.504 |  | 0.0872 |  | 0.333 |  | 0.675 |  | 5.77 |  | < .001 |  |
| Negative Urgency |  | → |  | BAQ12 |  | b |  | 2.164 |  | 0.3364 |  | 1.505 |  | 2.823 |  | 6.43 |  | < .001 |  |
| Relationships |  | → |  | BAQ12 |  | c |  | 0.518 |  | 0.3784 |  | -0.224 |  | 1.259 |  | 1.37 |  | 0.171 |  |
|  | | | | | | | | | | | | | | | | | | | |

Self Image via Negative urgency to BAQ12

**Mediation**

| Mediation Estimates | | | | | | | | | | | | | | | | | | |
| --- | --- | --- | --- | --- | --- | --- | --- | --- | --- | --- | --- | --- | --- | --- | --- | --- | --- | --- |
|  | | | | | | | | **95% Confidence Interval** | | | |  | | | | | | |
| **Effect** | | **Label** | | **Estimate** | | **SE** | | **Lower** | | **Upper** | | **Z** | | **P** | **P’** | | **% Mediation** | |
| Indirect |  | a × b |  | 0.643 |  | 0.261 |  | 0.1324 |  | 1.15 |  | 2.47 |  | 0.014 | | 0.05 | 43.8 |  |
| Direct |  | c |  | 0.825 |  | 0.398 |  | 0.0452 |  | 1.60 |  | 2.07 |  | 0.038 | | 0.19 | 56.2 |  |
| Total |  | c + a × b |  | 1.468 |  | 0.459 |  | 0.5686 |  | 2.37 |  | 3.20 |  | 0.001 | | **0.005** | 100.0 |  |
| Note : P´= Bonferroni correction of p, Bonferroni alpha’ = .01 from alpha=0.05 | | | | | | | | | | | | | | | | | | |

| Path Estimates | | | | | | | | | | | | | | | | | | | |
| --- | --- | --- | --- | --- | --- | --- | --- | --- | --- | --- | --- | --- | --- | --- | --- | --- | --- | --- | --- |
|  | | | | | | | | | | | | **95% Confidence Interval** | | | |  | | | |
|  | |  | |  | | **Label** | | **Estimate** | | **SE** | | **Lower** | | **Upper** | | **Z** | | **p** | |
| Self Image |  | → |  | Negative Urgency |  | a |  | 0.288 |  | 0.110 |  | 0.0729 |  | 0.504 |  | 2.62 |  | 0.009 |  |
| Negative Urgency |  | → |  | BAQ12 |  | b |  | 2.229 |  | 0.306 |  | 1.6302 |  | 2.829 |  | 7.29 |  | < .001 |  |
| Self Image |  | → |  | BAQ12 |  | c |  | 0.825 |  | 0.398 |  | 0.0452 |  | 1.604 |  | 2.07 |  | 0.038 |  |
|  | | | | | | | | | | | | | | | | | | | |

Suicide via Negative urgency to BAQ12

**Mediation**

| Mediation Estimates | | | | | | | | | | | | | | | | | | |
| --- | --- | --- | --- | --- | --- | --- | --- | --- | --- | --- | --- | --- | --- | --- | --- | --- | --- | --- |
|  | | | | | | | | **95% Confidence Interval** | | | |  | | | | | | |
| **Effect** | | **Label** | | **Estimate** | | **SE** | | **Lower** | | **Upper** | | **Z** | | **p** | **P’** | | **% Mediation** | |
| Indirect |  | a × b |  | 0.103 |  | 0.231 |  | -0.350 |  | 0.555 |  | 0.444 |  | 0.657 | | 3.285 | 25.4 |  |
| Direct |  | c |  | 0.301 |  | 0.341 |  | -0.368 |  | 0.969 |  | 0.881 |  | 0.378 | | 1.89 | 74.6 |  |
| Total |  | c + a × b |  | 0.403 |  | 0.412 |  | -0.404 |  | 1.210 |  | 0.979 |  | 0.327 | | 1.635 | 100.0 |  |
| Note : P´= Bonferroni correction of p, Bonferroni alpha’ = .01 from alpha=0.05 | | | | | | | | | | | | | | | | | | |

| Path Estimates | | | | | | | | | | | | | | | | | | | |
| --- | --- | --- | --- | --- | --- | --- | --- | --- | --- | --- | --- | --- | --- | --- | --- | --- | --- | --- | --- |
|  | | | | | | | | | | | | **95% Confidence Interval** | | | |  | | | |
|  | |  | |  | | **Label** | | **Estimate** | | **SE** | | **Lower** | | **Upper** | | **Z** | | **p** | |
| Suicide |  | → |  | Negative Urgency |  | a |  | 0.0434 |  | 0.0978 |  | -0.148 |  | 0.235 |  | 0.444 |  | 0.657 |  |
| Negative Urgency |  | → |  | BAQ12 |  | b |  | 2.3598 |  | 0.3023 |  | 1.767 |  | 2.952 |  | 7.807 |  | < .001 |  |
| Suicide |  | → |  | BAQ12 |  | c |  | 0.3006 |  | 0.3411 |  | -0.368 |  | 0.969 |  | 0.881 |  | 0.378 |  |
|  | | | | | | | | | | | | | | | | | | | |

Emptiness via Negative urgency to BAQ12

**Mediation**

| Mediation Estimates | | | | | | | | | | | | | | | | | | |
| --- | --- | --- | --- | --- | --- | --- | --- | --- | --- | --- | --- | --- | --- | --- | --- | --- | --- | --- |
|  | | | | | | | | **95% Confidence Interval** | | | |  | | | | | | |
| **Effect** | | **Label** | | **Estimate** | | **SE** | | **Lower** | | **Upper** | | **Z** | | **p** | **P’** | | **% Mediation** | |
| Indirect |  | a × b |  | 0.662 |  | 0.199 |  | 0.271 |  | 1.05 |  | 3.32 |  | < .001 | | <.005 | 34.3 |  |
| Direct |  | c |  | 1.265 |  | 0.318 |  | 0.642 |  | 1.89 |  | 3.98 |  | < .001 | | <.005 | 65.7 |  |
| Total |  | c + a × b |  | 1.927 |  | 0.347 |  | 1.246 |  | 2.61 |  | 5.55 |  | < .001 | | **<.005** | 100.0 |  |
| Note : P´= Bonferroni correction of p, Bonferroni alpha’ = .01 from alpha=0.05 | | | | | | | | | | | | | | | | | | |

| Path Estimates | | | | | | | | | | | | | | | | | | | |
| --- | --- | --- | --- | --- | --- | --- | --- | --- | --- | --- | --- | --- | --- | --- | --- | --- | --- | --- | --- |
|  | | | | | | | | | | | | **95% Confidence Interval** | | | |  | | | |
|  | |  | |  | | **Label** | | **Estimate** | | **SE** | | **Lower** | | **Upper** | | **Z** | | **p** | |
| Emptiness |  | → |  | Negative Urgency |  | a |  | 0.332 |  | 0.0866 |  | 0.163 |  | 0.502 |  | 3.84 |  | < .001 |  |
| Negative Urgency |  | → |  | BAQ12 |  | b |  | 1.990 |  | 0.3018 |  | 1.399 |  | 2.582 |  | 6.60 |  | < .001 |  |
| Emptiness |  | → |  | BAQ12 |  | c |  | 1.265 |  | 0.3176 |  | 0.642 |  | 1.887 |  | 3.98 |  | < .001 |  |
|  | | | | | | | | | | | | | | | | | | | |

Intense Anger via Negative urgency to BAQ12

**Mediation**

| Mediation Estimates | | | | | | | | | | | | | | | | | | |
| --- | --- | --- | --- | --- | --- | --- | --- | --- | --- | --- | --- | --- | --- | --- | --- | --- | --- | --- |
|  | | | | | | | | **95% Confidence Interval** | | | |  | | | | | | |
| **Effect** | | **Label** | | **Estimate** | | **SE** | | **Lower** | | **Upper** | | **Z** | | **p** | **P’** | | **% Mediation** | |
| Indirect |  | a × b |  | 0.710 |  | 0.196 |  | 0.325 |  | 1.09 |  | 3.61 |  | < .001 | | <.005 | 26.6 |  |
| Direct |  | c |  | 1.956 |  | 0.318 |  | 1.333 |  | 2.58 |  | 6.16 |  | < .001 | | <.005 | 73.4 |  |
| Total |  | c + a × b |  | 2.665 |  | 0.284 |  | 2.109 |  | 3.22 |  | 9.39 |  | < .001 | | **<.005** | 100.0 |  |
| Note : P´= Bonferroni correction of p, Bonferroni alpha’ = .01 from alpha=0.05 | | | | | | | | | | | | | | | | | | |

| Path Estimates | | | | | | | | | | | | | | | | | | | |
| --- | --- | --- | --- | --- | --- | --- | --- | --- | --- | --- | --- | --- | --- | --- | --- | --- | --- | --- | --- |
|  | | | | | | | | | | | | **95% Confidence Interval** | | | |  | | | |
|  | |  | |  | | **Label** | | **Estimate** | | **SE** | | **Lower** | | **Upper** | | **Z** | | **p** | |
| Intense Anger |  | → |  | Negative Urgency |  | a |  | 0.540 |  | 0.0730 |  | 0.397 |  | 0.683 |  | 7.40 |  | < .001 |  |
| Negative Urgency |  | → |  | BAQ12 |  | b |  | 1.314 |  | 0.3175 |  | 0.692 |  | 1.936 |  | 4.14 |  | < .001 |  |
| Intense Anger |  | → |  | BAQ12 |  | c |  | 1.956 |  | 0.3175 |  | 1.333 |  | 2.578 |  | 6.16 |  | < .001 |  |
|  | | | | | | | | | | | | | | | | | | | |

Psychotic via Negative urgency to BAQ12

**Mediation**

| Mediation Estimates | | | | | | | | | | | | | | | | | | |
| --- | --- | --- | --- | --- | --- | --- | --- | --- | --- | --- | --- | --- | --- | --- | --- | --- | --- | --- |
|  | | | | | | | | **95% Confidence Interval** | | | |  | | | | | | |
| **Effect** | | **Label** | | **Estimate** | | **SE** | | **Lower** | | **Upper** | | **Z** | | **p** | **P’** | | **% Mediation** | |
| Indirect |  | a × b |  | 0.993 |  | 0.293 |  | 0.419 |  | 1.57 |  | 3.39 |  | < .001 | | <.005 | 49.8 |  |
| Direct |  | c |  | 1.003 |  | 0.453 |  | 0.115 |  | 1.89 |  | 2.22 |  | 0.027 | | .135 | 50.2 |  |
| Total |  | c + a × b |  | 1.996 |  | 0.498 |  | 1.019 |  | 2.97 |  | 4.00 |  | < .001 | | **<.005** | 100.0 |  |
| Note : P´= Bonferroni correction of p, Bonferroni alpha’ = .01 from alpha=0.05 | | | | | | | | | | | | | | | | | | |

| Path Estimates | | | | | | | | | | | | | | | | | | | |
| --- | --- | --- | --- | --- | --- | --- | --- | --- | --- | --- | --- | --- | --- | --- | --- | --- | --- | --- | --- |
|  | | | | | | | | | | | | **95% Confidence Interval** | | | |  | | | |
|  | |  | |  | | **Label** | | **Estimate** | | **SE** | | **Lower** | | **Upper** | | **Z** | | **p** | |
| Quasi Psychotic |  | → |  | Negative Urgency |  | a |  | 0.462 |  | 0.118 |  | 0.231 |  | 0.694 |  | 3.91 |  | < .001 |  |
| Negative Urgency |  | → |  | BAQ12 |  | b |  | 2.147 |  | 0.314 |  | 1.531 |  | 2.762 |  | 6.83 |  | < .001 |  |
| Quasi Psychotic |  | → |  | BAQ12 |  | c |  | 1.003 |  | 0.453 |  | 0.115 |  | 1.890 |  | 2.22 |  | 0.027 |  |
|  | | | | | | | | | | | | | | | | | | | |

**Impulsivity via Positive Urgency to BAQ12**

**Mediation**

| Mediation Estimates | | | | | | | | | | | | | | | | | | |
| --- | --- | --- | --- | --- | --- | --- | --- | --- | --- | --- | --- | --- | --- | --- | --- | --- | --- | --- |
|  | | | | | | | | **95% Confidence Interval** | | | |  | | | | | | |
| **Effect** | | **Label** | | **Estimate** | | **SE** | | **Lower** | | **Upper** | | **Z** | | **p** | **P’** | | **% Mediation** | |
| Indirect |  | a × b |  | 1.01 |  | 0.340 |  | 0.3474 |  | 1.68 |  | 2.98 |  | 0.003 | | .015 | 46.9 |  |
| Direct |  | c |  | 1.15 |  | 0.561 |  | 0.0490 |  | 2.25 |  | 2.05 |  | 0.041 | | .205 | 53.1 |  |
| Total |  | c + a × b |  | 2.16 |  | 0.483 |  | 1.2163 |  | 3.11 |  | 4.48 |  | < .001 | | **<.005** | 100.0 |  |
| Note : P´= Bonferroni correction of p, Bonferroni alpha’ = .01 from alpha=0.05 | | | | | | | | | | | | | | | | | | |

| Path Estimates | | | | | | | | | | | | | | | | | | | |
| --- | --- | --- | --- | --- | --- | --- | --- | --- | --- | --- | --- | --- | --- | --- | --- | --- | --- | --- | --- |
|  | | | | | | | | | | | | **95% Confidence Interval** | | | |  | | | |
|  | |  | |  | | **Label** | | **Estimate** | | **SE** | | **Lower** | | **Upper** | | **Z** | | **p** | |
| Impulsivity |  | → |  | Positive Urgency |  | a |  | 0.773 |  | 0.0993 |  | 0.5783 |  | 0.967 |  | 7.79 |  | < .001 |  |
| Positive Urgency |  | → |  | BAQ12 |  | b |  | 1.312 |  | 0.4065 |  | 0.5153 |  | 2.109 |  | 3.23 |  | 0.001 |  |
| Impulsivity |  | → |  | BAQ12 |  | c |  | 1.149 |  | 0.5615 |  | 0.0490 |  | 2.250 |  | 2.05 |  | 0.041 |  |
|  | | | | | | | | | | | | | | | | | | | |

Affective instability via Positive Urgency to BAQ12

**Mediation**

| Mediation Estimates | | | | | | | | | | | | | | | | | | |
| --- | --- | --- | --- | --- | --- | --- | --- | --- | --- | --- | --- | --- | --- | --- | --- | --- | --- | --- |
|  | | | | | | | | **95% Confidence Interval** | | | |  | | | | | | |
| **Effect** | | **Label** | | **Estimate** | | **SE** | | **Lower** | | **Upper** | | **Z** | | **p** | **P’** | | **% Mediation** | |
| Indirect |  | a × b |  | 0.486 |  | 0.174 |  | 0.146 |  | 0.826 |  | 2.80 |  | 0.005 | | .025 | 24.9 |  |
| Direct |  | c |  | 1.469 |  | 0.378 |  | 0.728 |  | 2.211 |  | 3.88 |  | < .001 | | <.005 | 75.1 |  |
| Total |  | c + a × b |  | 1.955 |  | 0.377 |  | 1.216 |  | 2.695 |  | 5.18 |  | < .001 | | **<.005** | 100.0 |  |
| Note : P´= Bonferroni correction of p, Bonferroni alpha’ = .01 from alpha=0.05 | | | | | | | | | | | | | | | | | | |

| Path Estimates | | | | | | | | | | | | | | | | | | | |
| --- | --- | --- | --- | --- | --- | --- | --- | --- | --- | --- | --- | --- | --- | --- | --- | --- | --- | --- | --- |
|  | | | | | | | | | | | | **95% Confidence Interval** | | | |  | | | |
|  | |  | |  | | **Label** | | **Estimate** | | **SE** | | **Lower** | | **Upper** | | **Z** | | **p** | |
| Affect instability |  | → |  | Positive Urgency |  | a |  | 0.363 |  | 0.0902 |  | 0.186 |  | 0.540 |  | 4.03 |  | < .001 |  |
| Positive Urgency |  | → |  | BAQ12 |  | b |  | 1.338 |  | 0.3435 |  | 0.665 |  | 2.011 |  | 3.90 |  | < .001 |  |
| Affect instability |  | → |  | BAQ12 |  | c |  | 1.469 |  | 0.3785 |  | 0.728 |  | 2.211 |  | 3.88 |  | < .001 |  |
|  | | | | | | | | | | | | | | | | | | | |

Abandonment via Positive Urgency to BAQ12

**Mediation**

| Mediation Estimates | | | | | | | | | | | | | | | | | | |
| --- | --- | --- | --- | --- | --- | --- | --- | --- | --- | --- | --- | --- | --- | --- | --- | --- | --- | --- |
|  | | | | | | | | **95% Confidence Interval** | | | |  | | | | | | |
| **Effect** | | **Label** | | **Estimate** | | **SE** | | **Lower** | | **Upper** | | **Z** | | **p** | **P’** | | **% Mediation** | |
| Indirect |  | a × b |  | 0.403 |  | 0.169 |  | 0.0712 |  | 0.735 |  | 2.38 |  | 0.017 | | .085 | 18.6 |  |
| Direct |  | c |  | 1.762 |  | 0.388 |  | 1.0015 |  | 2.522 |  | 4.54 |  | < .001 | | <.005 | 81.4 |  |
| Total |  | c + a × b |  | 2.165 |  | 0.402 |  | 1.3764 |  | 2.953 |  | 5.38 |  | < .001 | | **<.005** | 100.0 |  |
| Note : P´= Bonferroni correction of p, Bonferroni alpha’ = .01 from alpha=0.05 | | | | | | | | | | | | | | | | | | |

| Path Estimates | | | | | | | | | | | | | | | | | | | |
| --- | --- | --- | --- | --- | --- | --- | --- | --- | --- | --- | --- | --- | --- | --- | --- | --- | --- | --- | --- |
|  | | | | | | | | | | | | **95% Confidence Interval** | | | |  | | | |
|  | |  | |  | | **Label** | | **Estimate** | | **SE** | | **Lower** | | **Upper** | | **Z** | | **p** | |
| Abandonement |  | → |  | Positive Urgency |  | a |  | 0.284 |  | 0.0996 |  | 0.0886 |  | 0.479 |  | 2.85 |  | 0.004 |  |
| Positive Urgency |  | → |  | BAQ12 |  | b |  | 1.420 |  | 0.3280 |  | 0.7776 |  | 2.063 |  | 4.33 |  | < .001 |  |
| Abandonement |  | → |  | BAQ12 |  | c |  | 1.762 |  | 0.3879 |  | 1.0015 |  | 2.522 |  | 4.54 |  | < .001 |  |
|  | | | | | | | | | | | | | | | | | | | |

Relationships via Positive Urgency to BAQ12

**Mediation**

| Mediation Estimates | | | | | | | | | | | | | | | | | | |
| --- | --- | --- | --- | --- | --- | --- | --- | --- | --- | --- | --- | --- | --- | --- | --- | --- | --- | --- |
|  | | | | | | | | **95% Confidence Interval** | | | |  | | | | | | |
| **Effect** | | **Label** | | **Estimate** | | **SE** | | **Lower** | | **Upper** | | **Z** | | **p** | **P’** | | **% Mediation** | |
| Indirect |  | a × b |  | 0.523 |  | 0.182 |  | 0.166 |  | 0.879 |  | 2.87 |  | 0.004 | | .020 | 32.5 |  |
| Direct |  | c |  | 1.084 |  | 0.386 |  | 0.328 |  | 1.841 |  | 2.81 |  | 0.005 | | .025 | 67.5 |  |
| Total |  | c + a × b |  | 1.607 |  | 0.388 |  | 0.848 |  | 2.367 |  | 4.15 |  | < .001 | | **<.005** | 100.0 |  |
| Note : P´= Bonferroni correction of p, Bonferroni alpha’ = .01 from alpha=0.05 | | | | | | | | | | | | | | | | | | |

| Path Estimates | | | | | | | | | | | | | | | | | | | |
| --- | --- | --- | --- | --- | --- | --- | --- | --- | --- | --- | --- | --- | --- | --- | --- | --- | --- | --- | --- |
|  | | | | | | | | | | | | **95% Confidence Interval** | | | |  | | | |
|  | |  | |  | | **Label** | | **Estimate** | | **SE** | | **Lower** | | **Upper** | | **Z** | | **p** | |
| Relationships |  | → |  | Positive Urgency |  | a |  | 0.360 |  | 0.0899 |  | 0.183 |  | 0.536 |  | 4.00 |  | < .001 |  |
| Positive Urgency |  | → |  | BAQ12 |  | b |  | 1.454 |  | 0.3519 |  | 0.764 |  | 2.144 |  | 4.13 |  | < .001 |  |
| Relationships |  | → |  | BAQ12 |  | c |  | 1.084 |  | 0.3861 |  | 0.328 |  | 1.841 |  | 2.81 |  | 0.005 |  |
|  | | | | | | | | | | | | | | | | | | | |

Self Image via Positive Urgency to BAQ12

**Mediation**

| Mediation Estimates | | | | | | | | | | | | | | | | | | |
| --- | --- | --- | --- | --- | --- | --- | --- | --- | --- | --- | --- | --- | --- | --- | --- | --- | --- | --- |
|  | | | | | | | | **95% Confidence Interval** | | | |  | | | | | | |
| **Effect** | | **Label** | | **Estimate** | | **SE** | | **Lower** | | **Upper** | | **Z** | | **p** | **P’** | | **% Mediation** | |
| Indirect |  | a × b |  | 0.177 |  | 0.189 |  | -0.194 |  | 0.547 |  | 0.936 |  | 0.349 | | 1.175 | 12.0 |  |
| Direct |  | c |  | 1.291 |  | 0.421 |  | 0.466 |  | 2.116 |  | 3.067 |  | 0.002 | | .010 | 88.0 |  |
| Total |  | c + a × b |  | 1.468 |  | 0.459 |  | 0.569 |  | 2.367 |  | 3.199 |  | 0.001 | | **.005** | 100.0 |  |
| Note : P´= Bonferroni correction of p, Bonferroni alpha’ = .01 from alpha=0.05 | | | | | | | | | | | | | | | | | | |

| Path Estimates | | | | | | | | | | | | | | | | | | | |
| --- | --- | --- | --- | --- | --- | --- | --- | --- | --- | --- | --- | --- | --- | --- | --- | --- | --- | --- | --- |
|  | | | | | | | | | | | | **95% Confidence Interval** | | | |  | | | |
|  | |  | |  | | **Label** | | **Estimate** | | **SE** | | **Lower** | | **Upper** | | **Z** | | **p** | |
| Self Image |  | → |  | Positive Urgency |  | a |  | 0.104 |  | 0.110 |  | -0.111 |  | 0.319 |  | 0.952 |  | 0.341 |  |
| Positive Urgency |  | → |  | BAQ12 |  | b |  | 1.694 |  | 0.332 |  | 1.044 |  | 2.344 |  | 5.105 |  | < .001 |  |
| Self Image |  | → |  | BAQ12 |  | c |  | 1.291 |  | 0.421 |  | 0.466 |  | 2.116 |  | 3.067 |  | 0.002 |  |
|  | | | | | | | | | | | | | | | | | | | |

Suicide via Positive Urgency to BAQ12

**Mediation**

| Mediation Estimates | | | | | | | | | | | | | | | | | | |
| --- | --- | --- | --- | --- | --- | --- | --- | --- | --- | --- | --- | --- | --- | --- | --- | --- | --- | --- |
|  | | | | | | | | **95% Confidence Interval** | | | |  | | | | | | |
| **Effect** | | **Label** | | **Estimate** | | **SE** | | **Lower** | | **Upper** | | **Z** | | **p** | **P’** | | **% Mediation** | |
| Indirect |  | a × b |  | -0.0414 |  | 0.171 |  | -0.376 |  | 0.293 |  | -0.243 |  | 0.808 | | 4.04 | 8.53 |  |
| Direct |  | c |  | 0.4446 |  | 0.375 |  | -0.290 |  | 1.179 |  | 1.186 |  | 0.235 | | 1.175 | 91.47 |  |
| Total |  | c + a × b |  | 0.4031 |  | 0.412 |  | -0.404 |  | 1.210 |  | 0.979 |  | 0.327 | | 1.635 | 100.00 |  |
| Note : P´= Bonferroni correction of p, Bonferroni alpha’ = .01 from alpha=0.05 | | | | | | | | | | | | | | | | | | |

| Path Estimates | | | | | | | | | | | | | | | | | | | |
| --- | --- | --- | --- | --- | --- | --- | --- | --- | --- | --- | --- | --- | --- | --- | --- | --- | --- | --- | --- |
|  | | | | | | | | | | | | **95% Confidence Interval** | | | |  | | | |
|  | |  | |  | | **Label** | | **Estimate** | | **SE** | | **Lower** | | **Upper** | | **Z** | | **p** | |
| Suicide |  | → |  | Positive Urgency |  | a |  | -0.0232 |  | 0.0954 |  | -0.210 |  | 0.164 |  | -0.243 |  | 0.808 |  |
| Positive Urgency |  | → |  | BAQ12 |  | b |  | 1.7862 |  | 0.3405 |  | 1.119 |  | 2.454 |  | 5.246 |  | < .001 |  |
| Suicide |  | → |  | BAQ12 |  | c |  | 0.4446 |  | 0.3747 |  | -0.290 |  | 1.179 |  | 1.186 |  | 0.235 |  |
|  | | | | | | | | | | | | | | | | | | | |

Emptiness via Positive Urgency to BAQ12

**Mediation**

| Mediation Estimates | | | | | | | | | | | | | | | | | | |
| --- | --- | --- | --- | --- | --- | --- | --- | --- | --- | --- | --- | --- | --- | --- | --- | --- | --- | --- |
|  | | | | | | | | **95% Confidence Interval** | | | |  | | | | | | |
| **Effect** | | **Label** | | **Estimate** | | **SE** | | **Lower** | | **Upper** | | **Z** | | **p** | **P’** | | **% Mediation** | |
| Indirect |  | a × b |  | 0.175 |  | 0.145 |  | -0.109 |  | 0.460 |  | 1.21 |  | 0.228 | | 1.14 | 9.10 |  |
| Direct |  | c |  | 1.751 |  | 0.319 |  | 1.126 |  | 2.377 |  | 5.49 |  | < .001 | | <.005 | 90.90 |  |
| Total |  | c + a × b |  | 1.927 |  | 0.347 |  | 1.246 |  | 2.607 |  | 5.55 |  | < .001 | | **<.005** | 100.00 |  |
| Note : P´= Bonferroni correction of p, Bonferroni alpha’ = .01 from alpha=0.05 | | | | | | | | | | | | | | | | | | |

| Path Estimates | | | | | | | | | | | | | | | | | | | |
| --- | --- | --- | --- | --- | --- | --- | --- | --- | --- | --- | --- | --- | --- | --- | --- | --- | --- | --- | --- |
|  | | | | | | | | | | | | **95% Confidence Interval** | | | |  | | | |
|  | |  | |  | | **Label** | | **Estimate** | | **SE** | | **Lower** | | **Upper** | | **Z** | | **p** | |
| Emptiness |  | → |  | Positive Urgency |  | a |  | 0.110 |  | 0.0885 |  | -0.0636 |  | 0.283 |  | 1.24 |  | 0.214 |  |
| Positive Urgency |  | → |  | BAQ12 |  | b |  | 1.595 |  | 0.3107 |  | 0.9861 |  | 2.204 |  | 5.13 |  | < .001 |  |
| Emptiness |  | → |  | BAQ12 |  | c |  | 1.751 |  | 0.3190 |  | 1.1262 |  | 2.377 |  | 5.49 |  | < .001 |  |
|  | | | | | | | | | | | | | | | | | | | |

Intense Anger via Positive Urgency to BAQ12

**Mediation**

| Mediation Estimates | | | | | | | | | | | | | | | | | | |
| --- | --- | --- | --- | --- | --- | --- | --- | --- | --- | --- | --- | --- | --- | --- | --- | --- | --- | --- |
|  | | | | | | | | **95% Confidence Interval** | | | |  | | | | | | |
| **Effect** | | **Label** | | **Estimate** | | **SE** | | **Lower** | | **Upper** | | **Z** | | **p** | **P’** | | **% Mediation** | |
| Indirect |  | a × b |  | 0.182 |  | 0.197 |  | -0.205 |  | 0.569 |  | 0.923 |  | 0.356 | | 1.78 | 6.84 |  |
| Direct |  | c |  | 2.483 |  | 0.344 |  | 1.808 |  | 3.158 |  | 7.213 |  | < .001 | | <.005 | 93.16 |  |
| Total |  | c + a × b |  | 2.665 |  | 0.284 |  | 2.109 |  | 3.222 |  | 9.388 |  | < .001 | | **<.005** | 100.00 |  |
| Note : P´= Bonferroni correction of p, Bonferroni alpha’ = .01 from alpha=0.05 | | | | | | | | | | | | | | | | | | |

| Path Estimates | | | | | | | | | | | | | | | | | | | |
| --- | --- | --- | --- | --- | --- | --- | --- | --- | --- | --- | --- | --- | --- | --- | --- | --- | --- | --- | --- |
|  | | | | | | | | | | | | **95% Confidence Interval** | | | |  | | | |
|  | |  | |  | | **Label** | | **Estimate** | | **SE** | | **Lower** | | **Upper** | | **Z** | | **p** | |
| Intense Anger |  | → |  | Positive Urgency |  | a |  | 0.556 |  | 0.0695 |  | 0.419 |  | 0.692 |  | 7.989 |  | < .001 |  |
| Positive Urgency |  | → |  | BAQ12 |  | b |  | 0.328 |  | 0.3529 |  | -0.364 |  | 1.020 |  | 0.930 |  | 0.353 |  |
| Intense Anger |  | → |  | BAQ12 |  | c |  | 2.483 |  | 0.3443 |  | 1.808 |  | 3.158 |  | 7.213 |  | < .001 |  |
|  | | | | | | | | | | | | | | | | | | | |

Psychotic via Positive Urgency to BAQ12

**Mediation**

| Mediation Estimates | | | | | | | | | | | | | | | | | | |
| --- | --- | --- | --- | --- | --- | --- | --- | --- | --- | --- | --- | --- | --- | --- | --- | --- | --- | --- |
|  | | | | | | | | **95% Confidence Interval** | | | |  | | | | | | |
| **Effect** | | **Label** | | **Estimate** | | **SE** | | **Lower** | | **Upper** | | **Z** | | **p** | **P’** | | **% Mediation** | |
| Indirect |  | a × b |  | 0.508 |  | 0.213 |  | 0.0897 |  | 0.926 |  | 2.38 |  | 0.017 | | .085 | 25.4 |  |
| Direct |  | c |  | 1.488 |  | 0.478 |  | 0.5510 |  | 2.425 |  | 3.11 |  | 0.002 | | .10 | 74.6 |  |
| Total |  | c + a × b |  | 1.996 |  | 0.498 |  | 1.0188 |  | 2.972 |  | 4.00 |  | < .001 | | **<.005** | 100.0 |  |
| Note : P´= Bonferroni correction of p, Bonferroni alpha’ = .01 from alpha=0.05 | | | | | | | | | | | | | | | | | | |

| Path Estimates | | | | | | | | | | | | | | | | | | | |
| --- | --- | --- | --- | --- | --- | --- | --- | --- | --- | --- | --- | --- | --- | --- | --- | --- | --- | --- | --- |
|  | | | | | | | | | | | | **95% Confidence Interval** | | | |  | | | |
|  | |  | |  | | **Label** | | **Estimate** | | **SE** | | **Lower** | | **Upper** | | **Z** | | **p** | |
| Quasi Psychotic |  | → |  | Positive Urgency |  | a |  | 0.332 |  | 0.118 |  | 0.100 |  | 0.565 |  | 2.81 |  | 0.005 |  |
| Positive Urgency |  | → |  | BAQ12 |  | b |  | 1.527 |  | 0.340 |  | 0.861 |  | 2.194 |  | 4.49 |  | < .001 |  |
| Quasi Psychotic |  | → |  | BAQ12 |  | c |  | 1.488 |  | 0.478 |  | 0.551 |  | 2.425 |  | 3.11 |  | 0.002 |  |
|  | | | | | | | | | | | | | | | | | | | |

**Impulsivity via Sensation Seeking to BAQ12**

**Mediation**

| Mediation Estimates | | | | | | | | | | | | | | | | | | |
| --- | --- | --- | --- | --- | --- | --- | --- | --- | --- | --- | --- | --- | --- | --- | --- | --- | --- | --- |
|  | | | | | | | | **95% Confidence Interval** | | | |  | | | | | | |
| **Effect** | | **Label** | | **Estimate** | | **SE** | | **Lower** | | **Upper** | | **Z** | | **p** | **P’** | | **% Mediation** | |
| Indirect |  | a × b |  | -0.0187 |  | 0.0622 |  | -0.141 |  | 0.103 |  | -0.301 |  | 0.763 | | 3.815 | 0.921 |  |
| Direct |  | c |  | 2.0144 |  | 0.4949 |  | 1.044 |  | 2.984 |  | 4.071 |  | < .001 | | <.005 | 99.079 |  |
| Total |  | c + a × b |  | 1.9956 |  | 0.4984 |  | 1.019 |  | 2.972 |  | 4.004 |  | < .001 | | **<.005** | 100.000 |  |
| Note : P´= Bonferroni correction of p, Bonferroni alpha’ = .01 from alpha=0.05 | | | | | | | | | | | | | | | | | | |

| Path Estimates | | | | | | | | | | | | | | | | | | | |
| --- | --- | --- | --- | --- | --- | --- | --- | --- | --- | --- | --- | --- | --- | --- | --- | --- | --- | --- | --- |
|  | | | | | | | | | | | | **95% Confidence Interval** | | | |  | | | |
|  | |  | |  | | **Label** | | **Estimate** | | **SE** | | **Lower** | | **Upper** | | **Z** | | **p** | |
| Quasi Psychotic |  | → |  | Lack of Perseverance |  | a |  | -0.0478 |  | 0.155 |  | -0.352 |  | 0.256 |  | -0.308 |  | 0.758 |  |
| Lack of Perseverance |  | → |  | BAQ12 |  | b |  | 0.3915 |  | 0.276 |  | -0.150 |  | 0.933 |  | 1.416 |  | 0.157 |  |
| Quasi Psychotic |  | → |  | BAQ12 |  | c |  | 2.0144 |  | 0.495 |  | 1.044 |  | 2.984 |  | 4.071 |  | < .001 |  |
|  | | | | | | | | | | | | | | | | | | | |

Affect instability via Sensation Seeking to BAQ12

**Mediation**

| Mediation Estimates | | | | | | | | | | | | | | | | | | |
| --- | --- | --- | --- | --- | --- | --- | --- | --- | --- | --- | --- | --- | --- | --- | --- | --- | --- | --- |
|  | | | | | | | | **95% Confidence Interval** | | | |  | | | | | | |
| **Effect** | | **Label** | | **Estimate** | | **SE** | | **Lower** | | **Upper** | | **Z** | | **p** | **P’** | | **% Mediation** | |
| Indirect |  | a × b |  | 0.112 |  | 0.0965 |  | -0.0776 |  | 0.301 |  | 1.16 |  | 0.248 | | 1.24 | 5.70 |  |
| Direct |  | c |  | 1.844 |  | 0.3691 |  | 1.1205 |  | 2.567 |  | 5.00 |  | < .001 | | <.005 | 94.30 |  |
| Total |  | c + a × b |  | 1.955 |  | 0.3771 |  | 1.2162 |  | 2.695 |  | 5.18 |  | < .001 | | **<.005** | 100.00 |  |
| Note : P´= Bonferroni correction of p, Bonferroni alpha’ = .01 from alpha=0.05 | | | | | | | | | | | | | | | | | | |

| Path Estimates | | | | | | | | | | | | | | | | | | | |
| --- | --- | --- | --- | --- | --- | --- | --- | --- | --- | --- | --- | --- | --- | --- | --- | --- | --- | --- | --- |
|  | | | | | | | | | | | | **95% Confidence Interval** | | | |  | | | |
|  | |  | |  | | **Label** | | **Estimate** | | **SE** | | **Lower** | | **Upper** | | **Z** | | **p** | |
| Affect instability |  | → |  | Sensation Seeking |  | a |  | 0.148 |  | 0.116 |  | -0.0799 |  | 0.376 |  | 1.27 |  | 0.203 |  |
| Sensation Seeking |  | → |  | BAQ12 |  | b |  | 0.753 |  | 0.273 |  | 0.2170 |  | 1.289 |  | 2.75 |  | 0.006 |  |
| Affect instability |  | → |  | BAQ12 |  | c |  | 1.844 |  | 0.369 |  | 1.1205 |  | 2.567 |  | 5.00 |  | < .001 |  |
|  | | | | | | | | | | | | | | | | | | | |

Abandonnement via Sensation Seeking to BAQ12

**Mediation**

| Mediation Estimates | | | | | | | | | | | | | | | | | | |
| --- | --- | --- | --- | --- | --- | --- | --- | --- | --- | --- | --- | --- | --- | --- | --- | --- | --- | --- |
|  | | | | | | | | **95% Confidence Interval** | | | |  | | | | | | |
| **Effect** | | **Label** | | **Estimate** | | **SE** | | **Lower** | | **Upper** | | **Z** | | **p** | **P’** | | **% Mediation** | |
| Indirect |  | a × b |  | 0.118 |  | 0.103 |  | -0.0840 |  | 0.319 |  | 1.14 |  | 0.253 | | 1.265 | 5.44 |  |
| Direct |  | c |  | 2.047 |  | 0.394 |  | 1.2759 |  | 2.819 |  | 5.20 |  | < .001 | | <.005 | 94.56 |  |
| Total |  | c + a × b |  | 2.165 |  | 0.402 |  | 1.3764 |  | 2.953 |  | 5.38 |  | < .001 | | **<.005** | 100.00 |  |
| Note : P´= Bonferroni correction of p, Bonferroni alpha’ = .01 from alpha=0.05 | | | | | | | | | | | | | | | | | | |

| Path Estimates | | | | | | | | | | | | | | | | | | | |
| --- | --- | --- | --- | --- | --- | --- | --- | --- | --- | --- | --- | --- | --- | --- | --- | --- | --- | --- | --- |
|  | | | | | | | | | | | | **95% Confidence Interval** | | | |  | | | |
|  | |  | |  | | **Label** | | **Estimate** | | **SE** | | **Lower** | | **Upper** | | **Z** | | **p** | |
| Abandonement |  | → |  | Sensation Seeking |  | a |  | 0.157 |  | 0.125 |  | -0.0879 |  | 0.402 |  | 1.26 |  | 0.209 |  |
| Sensation Seeking |  | → |  | BAQ12 |  | b |  | 0.750 |  | 0.272 |  | 0.2176 |  | 1.282 |  | 2.76 |  | 0.006 |  |
| Abandonement |  | → |  | BAQ12 |  | c |  | 2.047 |  | 0.394 |  | 1.2759 |  | 2.819 |  | 5.20 |  | < .001 |  |
|  | | | | | | | | | | | | | | | | | | | |

Relationships via Sensation Seeking to BAQ12

**Mediation**

| Mediation Estimates | | | | | | | | | | | | | | | | | |
| --- | --- | --- | --- | --- | --- | --- | --- | --- | --- | --- | --- | --- | --- | --- | --- | --- | --- |
|  | | | | | | | | **95% Confidence Interval** | | | |  | | | | | |
| **Effect** | | **Label** | | **Estimate** | | **SE** | | **Lower** | | **Upper** | | **Z** | | **p** | | **% Mediation** | |
| Indirect |  | a × b |  | 0.0587 |  | 0.100 |  | -0.137 |  | 0.255 |  | 0.586 |  | 0.558 | 2.79 | 3.65 |  |
| Direct |  | c |  | 1.5486 |  | 0.375 |  | 0.813 |  | 2.284 |  | 4.126 |  | < .001 | <.005 | 96.35 |  |
| Total |  | c + a × b |  | 1.6073 |  | 0.388 |  | 0.848 |  | 2.367 |  | 4.148 |  | < .001 | **<.005** | 100.00 |  |
| Note : P´= Bonferroni correction of p, Bonferroni alpha’ = .01 from alpha=0.05 | | | | | | | | | | | | | | | | | |

| Path Estimates | | | | | | | | | | | | | | | | | | | |
| --- | --- | --- | --- | --- | --- | --- | --- | --- | --- | --- | --- | --- | --- | --- | --- | --- | --- | --- | --- |
|  | | | | | | | | | | | | **95% Confidence Interval** | | | |  | | | |
|  | |  | |  | | **Label** | | **Estimate** | | **SE** | | **Lower** | | **Upper** | | **Z** | | **p** | |
| Relationships |  | → |  | Sensation Seeking |  | a |  | 0.0696 |  | 0.116 |  | -0.159 |  | 0.298 |  | 0.598 |  | 0.550 |  |
| Sensation Seeking |  | → |  | BAQ12 |  | b |  | 0.8431 |  | 0.279 |  | 0.296 |  | 1.390 |  | 3.020 |  | 0.003 |  |
| Relationships |  | → |  | BAQ12 |  | c |  | 1.5486 |  | 0.375 |  | 0.813 |  | 2.284 |  | 4.126 |  | < .001 |  |
|  | | | | | | | | | | | | | | | | | | | |

Self image via Sensation Seeking to BAQ12

**Mediation**

| Mediation Estimates | | | | | | | | | | | | | | | | | |
| --- | --- | --- | --- | --- | --- | --- | --- | --- | --- | --- | --- | --- | --- | --- | --- | --- | --- |
|  | | | | | | | | **95% Confidence Interval** | | | |  | | | | | |
| **Effect** | | **Label** | | **Estimate** | | **SE** | | **Lower** | | **Upper** | | **Z** | | **p** | | **% Mediation** | |
| Indirect |  | a × b |  | -0.142 |  | 0.140 |  | -0.416 |  | 0.132 |  | -1.01 |  | 0.310 | .155 | 8.10 |  |
| Direct |  | c |  | 1.610 |  | 0.441 |  | 0.746 |  | 2.473 |  | 3.65 |  | < .001 | <.005 | 91.90 |  |
| Total |  | c + a × b |  | 1.468 |  | 0.459 |  | 0.569 |  | 2.367 |  | 3.20 |  | 0.001 | **.005** | 100.00 |  |
| Note : P´= Bonferroni correction of p, Bonferroni alpha’ = .01 from alpha=0.05 | | | | | | | | | | | | | | | | | |

| Path Estimates | | | | | | | | | | | | | | | | | | | |
| --- | --- | --- | --- | --- | --- | --- | --- | --- | --- | --- | --- | --- | --- | --- | --- | --- | --- | --- | --- |
|  | | | | | | | | | | | | **95% Confidence Interval** | | | |  | | | |
|  | |  | |  | | **Label** | | **Estimate** | | **SE** | | **Lower** | | **Upper** | | **Z** | | **p** | |
| Self Image |  | → |  | Sensation Seeking |  | a |  | -0.142 |  | 0.134 |  | -0.405 |  | 0.121 |  | -1.06 |  | 0.289 |  |
| Sensation Seeking |  | → |  | BAQ12 |  | b |  | 0.997 |  | 0.283 |  | 0.442 |  | 1.553 |  | 3.52 |  | < .001 |  |
| Self Image |  | → |  | BAQ12 |  | c |  | 1.610 |  | 0.441 |  | 0.746 |  | 2.473 |  | 3.65 |  | < .001 |  |
|  | | | | | | | | | | | | | | | | | | | |

Suicide via Sensation Seeking to BAQ12

**Mediation**

| Mediation Estimates | | | | | | | | | | | | | | | | | | |
| --- | --- | --- | --- | --- | --- | --- | --- | --- | --- | --- | --- | --- | --- | --- | --- | --- | --- | --- |
|  | | | | | | | | **95% Confidence Interval** | | | |  | | | | | | |
| **Effect** | | **Label** | | **Estimate** | | **SE** | | **Lower** | | **Upper** | | **Z** | | **p** | **P’** | | **% Mediation** | |
| Indirect |  | a × b |  | 0.192 |  | 0.121 |  | -0.0445 |  | 0.428 |  | 1.591 |  | 0.112 | | .056 | 47.6 |  |
| Direct |  | c |  | 0.211 |  | 0.404 |  | -0.5812 |  | 1.004 |  | 0.522 |  | 0.601 | | 3.005 | 52.4 |  |
| Total |  | c + a × b |  | 0.403 |  | 0.412 |  | -0.4036 |  | 1.210 |  | 0.979 |  | 0.327 | | 1.635 | 100.0 |  |
| Note : P´= Bonferroni correction of p, Bonferroni alpha’ = .01 from alpha=0.05 | | | | | | | | | | | | | | | | | | |

| Path Estimates | | | | | | | | | | | | | | | | | | | |
| --- | --- | --- | --- | --- | --- | --- | --- | --- | --- | --- | --- | --- | --- | --- | --- | --- | --- | --- | --- |
|  | | | | | | | | | | | | **95% Confidence Interval** | | | |  | | | |
|  | |  | |  | | **Label** | | **Estimate** | | **SE** | | **Lower** | | **Upper** | | **Z** | | **p** | |
| Suicide |  | → |  | Sensation Seeking |  | a |  | 0.219 |  | 0.115 |  | -0.00741 |  | 0.445 |  | 1.896 |  | 0.058 |  |
| Sensation Seeking |  | → |  | BAQ12 |  | b |  | 0.877 |  | 0.300 |  | 0.28976 |  | 1.465 |  | 2.926 |  | 0.003 |  |
| Suicide |  | → |  | BAQ12 |  | c |  | 0.211 |  | 0.404 |  | -0.58118 |  | 1.004 |  | 0.522 |  | 0.601 |  |
|  | | | | | | | | | | | | | | | | | | | |

Emptiness via Sensation Seeking to BAQ12

**Mediation**

| Mediation Estimates | | | | | | | | | | | | | | | | | | |
| --- | --- | --- | --- | --- | --- | --- | --- | --- | --- | --- | --- | --- | --- | --- | --- | --- | --- | --- |
|  | | | | | | | | **95% Confidence Interval** | | | |  | | | | | | |
| **Effect** | | **Label** | | **Estimate** | | **SE** | | **Lower** | | **Upper** | | **Z** | | **p** | **P’** | | **% Mediation** | |
| Indirect |  | a × b |  | 0.0200 |  | 0.0956 |  | -0.167 |  | 0.207 |  | 0.209 |  | 0.835 | | 4.175 | 1.04 |  |
| Direct |  | c |  | 1.9066 |  | 0.3338 |  | 1.252 |  | 2.561 |  | 5.711 |  | < .001 | | <.005 | 98.96 |  |
| Total |  | c + a × b |  | 1.9266 |  | 0.3472 |  | 1.246 |  | 2.607 |  | 5.550 |  | < .001 | | **<.005** | 100.00 |  |
| Note : P´= Bonferroni correction of p, Bonferroni alpha’ = .01 from alpha=0.05 | | | | | | | | | | | | | | | | | | |

| Path Estimates | | | | | | | | | | | | | | | | | | | |
| --- | --- | --- | --- | --- | --- | --- | --- | --- | --- | --- | --- | --- | --- | --- | --- | --- | --- | --- | --- |
|  | | | | | | | | | | | | **95% Confidence Interval** | | | |  | | | |
|  | |  | |  | | **Label** | | **Estimate** | | **SE** | | **Lower** | | **Upper** | | **Z** | | **p** | |
| Emptiness |  | → |  | Sensation Seeking |  | a |  | 0.0228 |  | 0.109 |  | -0.191 |  | 0.237 |  | 0.209 |  | 0.834 |  |
| Sensation Seeking |  | → |  | BAQ12 |  | b |  | 0.8753 |  | 0.265 |  | 0.355 |  | 1.395 |  | 3.298 |  | < .001 |  |
| Emptiness |  | → |  | BAQ12 |  | c |  | 1.9066 |  | 0.334 |  | 1.252 |  | 2.561 |  | 5.711 |  | < .001 |  |
|  | | | | | | | | | | | | | | | | | | | |

Intense anger via Sensation Seeking to BAQ12

**Mediation**

| Mediation Estimates | | | | | | | | | | | | | | | | | |
| --- | --- | --- | --- | --- | --- | --- | --- | --- | --- | --- | --- | --- | --- | --- | --- | --- | --- |
|  | | | | | | | | **95% Confidence Interval** | | | |  | | | | | |
| **Effect** | | **Label** | | **Estimate** | | **SE** | | **Lower** | | **Upper** | | **Z** | | **p** | | **% Mediation** | |
| Indirect |  | a × b |  | 0.0915 |  | 0.0928 |  | -0.0904 |  | 0.273 |  | 0.986 |  | 0.324 | 1.62 | 3.43 |  |
| Direct |  | c |  | 2.5740 |  | 0.2966 |  | 1.9927 |  | 3.155 |  | 8.679 |  | < .001 | <.005 | 96.57 |  |
| Total |  | c + a × b |  | 2.6655 |  | 0.2839 |  | 2.1090 |  | 3.222 |  | 9.388 |  | < .001 | **<.005** | 100.00 |  |
| Note : P´= Bonferroni correction of p, Bonferroni alpha’ = .01 from alpha=0.05 | | | | | | | | | | | | | | | | | |

| Path Estimates | | | | | | | | | | | | | | | | | | | |
| --- | --- | --- | --- | --- | --- | --- | --- | --- | --- | --- | --- | --- | --- | --- | --- | --- | --- | --- | --- |
|  | | | | | | | | | | | | **95% Confidence Interval** | | | |  | | | |
|  | |  | |  | | **Label** | | **Estimate** | | **SE** | | **Lower** | | **Upper** | | **Z** | | **p** | |
| Intense Anger |  | → |  | Sensation Seeking |  | a |  | 0.360 |  | 0.0988 |  | 0.166 |  | 0.554 |  | 3.64 |  | < .001 |  |
| Sensation Seeking |  | → |  | BAQ12 |  | b |  | 0.254 |  | 0.2481 |  | -0.232 |  | 0.740 |  | 1.02 |  | 0.306 |  |
| Intense Anger |  | → |  | BAQ12 |  | c |  | 2.574 |  | 0.2966 |  | 1.993 |  | 3.155 |  | 8.68 |  | < .001 |  |
|  | | | | | | | | | | | | | | | | | | | |

Psychotic via Sensation Seeking to BAQ12

**Mediation**

| Mediation Estimates | | | | | | | | | | | | | | | | | | |
| --- | --- | --- | --- | --- | --- | --- | --- | --- | --- | --- | --- | --- | --- | --- | --- | --- | --- | --- |
|  | | | | | | | | **95% Confidence Interval** | | | |  | | | | | | |
| **Effect** | | **Label** | | **Estimate** | | **SE** | | **Lower** | | **Upper** | | **Z** | | **p** | **P’** | | **% Mediation** | |
| Indirect |  | a × b |  | 0.284 |  | 0.159 |  | -0.0275 |  | 0.595 |  | 1.79 |  | 0.074 | | .37 | 14.2 |  |
| Direct |  | c |  | 1.712 |  | 0.506 |  | 0.7202 |  | 2.703 |  | 3.38 |  | < .001 | | <.005 | 85.8 |  |
| Total |  | c + a × b |  | 1.996 |  | 0.498 |  | 1.0188 |  | 2.972 |  | 4.00 |  | < .001 | | **<.005** | 100.0 |  |
| Note : P´= Bonferroni correction of p, Bonferroni alpha’ = .01 from alpha=0.05 | | | | | | | | | | | | | | | | | | |

| Path Estimates | | | | | | | | | | | | | | | | | | | |
| --- | --- | --- | --- | --- | --- | --- | --- | --- | --- | --- | --- | --- | --- | --- | --- | --- | --- | --- | --- |
|  | | | | | | | | | | | | **95% Confidence Interval** | | | |  | | | |
|  | |  | |  | | **Label** | | **Estimate** | | **SE** | | **Lower** | | **Upper** | | **Z** | | **p** | |
| Quasi Psychotic |  | → |  | Sensation Seeking |  | a |  | 0.436 |  | 0.144 |  | 0.1527 |  | 0.719 |  | 3.02 |  | 0.003 |  |
| Sensation Seeking |  | → |  | BAQ12 |  | b |  | 0.651 |  | 0.294 |  | 0.0756 |  | 1.227 |  | 2.22 |  | 0.027 |  |
| Quasi Psychotic |  | → |  | BAQ12 |  | c |  | 1.712 |  | 0.506 |  | 0.7202 |  | 2.703 |  | 3.38 |  | < .001 |  |
|  | | | | | | | | | | | | | | | | | | | |
